# Barriers and Facilitators That Influence Telemedicine-Based, Real-Time, Online Consultation at Patients’ Homes: Systematic Literature Review

**DOI:** 10.2196/16407

**Published:** 2020-02-20

**Authors:** Hassan Khader Y Almathami, Khin Than Win, Elena Vlahu-Gjorgievska

**Affiliations:** 1 Faculty of Engineering and Information Sciences University of Wollongong Wollongong Australia; 2 College of Computers and Information Systems Umm Al-Qura University Makkah Saudi Arabia

**Keywords:** eHealth, mHealth, mobile health, video conferencing, electronic consultation, online consultation, facilitators, barriers

## Abstract

**Background:**

Health care providers are adopting information and communication technologies (ICTs) to enhance their services. Telemedicine is one of the services that rely heavily on ICTs to enable remote patients to communicate with health care professionals; in this case, the patient communicates with the health care professional for a follow-up or for a consultation about his or her health condition. This communication process is referred to as an e-consultation. In this paper, telemedicine services refer to health care services that use ICTs, which enable patients to share, transfer, and communicate data or information in real time (ie, synchronous) from their home with a care provider—normally a physician—at a clinical site. However, the use of e-consultation services can be positively or negatively influenced by external or internal factors. External factors refer to the environment surrounding the system as well as the system itself, while internal factors refer to user behavior and motivation.

**Objective:**

This review aims to investigate the barriers and the facilitators that influence the use of home consultation systems in the health care context. This review also aims to identify the effectiveness of Home Online Health Consultation (HOHC) systems in improving patients’ health as well as their satisfaction with the systems.

**Methods:**

We conducted a systematic literature review to search for articles—empirical studies—about online health consultation in four digital libraries: Scopus, Association for Computing Machinery, PubMed, and Web of Science. The database search yielded 2518 articles; after applying the inclusion and exclusion criteria, the number of included articles for the final review was 45. A qualitative content analysis was performed to identify barriers and facilitators to HOHC systems, their effectiveness, and patients’ satisfaction with them.

**Results:**

The systematic literature review identified several external and internal facilitators and barriers to HOHC systems that were used in the creation of a HOHC framework. The framework consists of four requirements; the framework also consists of 17 facilitators and eight barriers, which were further categorized as internal and external influencers on HOHC.

**Conclusions:**

Patients from different age groups and with different health conditions benefited from remote health services. HOHC via video conferencing was effective in delivering online treatment and was well-accepted by patients, as it simulated in-person, face-to-face consultation. Acceptance by patients increased as a result of online consultation facilitators that promoted effective and convenient remote treatment. However, some patients preferred face-to-face consultation and showed resistance to online consultation. Resistance to online consultation was influenced by some of the identified barriers. Overall, the framework identified the facilitators and barriers that positively and negatively influenced the uptake of HOHC systems, respectively.

## Introduction

Health care providers and professionals are using advanced information and communication technology (ICT) in telemedicine services to improve overall health care outcomes [1]. The World Health Organization describes telemedicine as “the delivery of health care services, where distance is a critical factor, by all health care professionals using information and communication technologies for the exchange of valid information for diagnosis, treatment and prevention of disease and injuries, research and evaluation, and for the continuing education of health care providers, all in the interest of advancing the health of individuals and their communities” [2]. Online electronic consultation is an important element of telemedicine, which is a service that relies heavily on ICTs; ICTs enable patients to communicate remotely with their care providers. Serrano and Karahanna [3] explained that “e-consultation refers to the telemedicine consultation session; the consulting expert is the consulting clinician (typically, a physician); and the remote client is a remote patient.” The patient communicates with a doctor for a follow-up or for a consultation about his or her health condition via video conferencing and telemedicine systems.

There are serval systematic literature reviews regarding the use of telemedicine and e-consultation in health care. Most of these studies are focused on telemedicine effectiveness, efficiency, and capability to improve health care services. Vimalananda et al [4] found that e-consultation between care providers improves patients’ access to specialty care without the need for face-to-face consultation by sharing patient records electronically in asynchronous mode between health care providers. Maarop and Win [5] found that a teleconsultation system that utilized the asynchronous store-and-forward method was considered an effective tool between Malaysian primary and tertiary health care facilities, due to the need for such services among health care providers and its perceived ease of use and usefulness. Roine et al [1] found that telemedicine technology provided an efficient and effective method of electronic referrals and video conferencing between primary and secondary health care providers, which saved health care services costs, especially in the transmission of computed tomography images and other services, such as teleradiology, teleneurosurgery, telepsychiatry, and transmission of echocardiographic reports. Similarly, Hasselberg et al [6] reported that image-based telemedicine systems for medical expert consultation in acute care of injuries provided valid diagnosis and influenced patient management by ensuring diagnostic validity, system quality, and satisfaction for clinicians and users. Caffery et al [7] found that telehealth interventions helped in reducing waiting times, waiting lists, and unnecessary appointments for patients who were seeking access to specialist outpatient services. Also, other systematic literature reviews investigated factors that influence the implementation, adaptably, sustainability, and acceptance of telemedicine services among health care professionals and health care providers [8-12].

Recently, telemedicine services have expanded from providing health care services at hospitals, outpatient departments, and specialist offices, as well as between health care providers, to deliver care at patients’ homes. For example, Vlahu-Gjorgievska et al [13] indicated utilizing a telemonitoring system for patients with congestive heart failure at home to improve their overall health condition, which would reduce their risk of hospitalization and rehospitalization due to its ability to empower a patient’s self-care, motivation, education, and self-management. Other studies indicated that telemedicine at patients’ homes provides remote health consultation, remote treatment, remote intervention, and remote assessment, which can improve patients’ health conditions at low cost [14,15]. Also, it eliminates patients’ waiting times, travel times, and travel expenses that occur when seeking face-to-face health consultation [16]. Further, it enables patients living in underserved areas to access health care specialists from the comfort of their homes [17].

Telemedicine at patients’ homes has been defined based on the type of the health care provided to the patient. Therefore, we define synchronous telemedicine health services as the Home Online Health Consultation (HOHC) system that enables patients to share, transfer, and communicate data or information in real time from their home with a care provider—normally a physician—at a clinical site, via telemedicine services that use ICTs.

The uptake of the HOHC system can be influenced by facilitators and barriers during its use. The facilitators refer to positive influencers, while barriers refer to negative influencers. Influencers can be either external or internal factors. External factors refer to the environment surrounding the system’s usage and the system itself, while internal factors refer to the user’s behavior and motivation while using the system. Therefore, there is a need to identify the facilitators and barriers to HOHC use.

The aim of this study is to provide an answer to our main question: (1) What are the facilitators and barriers to HOHC systems that influence their uptake? We also aim to provide answers to our subquestions: (2) Are HOHC systems effective? and (3) Are users satisfied with HOHC systems?

## Methods

### Study Design

We conducted a systematic literature review using four large databases to collect articles related to HOHC systems. We performed a qualitative content analysis to extract themes of facilitators and barriers to HOHC systems from each of the included articles.

### Step 1. Identification: Databases and Keywords

Four large digital libraries—Scopus, Association for Computing Machinery (ACM), PubMed, and Web of Science—were searched for articles about HOHC systems. We selected these databases because they include a large number of health journals. After identifying the digital libraries, with the help of a professional librarian, specific keywords were used to search for the needed articles from the identified databases. The keywords used in the search were as follows: (“eHealth” OR “health” OR “Telemedicine” OR “mHealth” OR “Mobile health”) AND (“video conferencing” OR “Electronic consultation” OR “online consultation” OR “e consultation”). The keywords were tested on Scopus, ACM, and Web of Science databases to check their validity with three constraints (ie, controls) on the search. The constraints were made to search for (1) articles only, (2) articles published in English, and (3) articles published from 2008 to 2018, as shown in Table 1. Further, PubMed was searched for (1) clinical trial and journal articles, (2) articles with human subjects, and (3) articles published in English in the last 10 years with the following Medical Subject Headings (MeSH) as keywords: “Telemedicine” AND “Remote Consultation.”

After the first test, several decisions were made: (1) to expand publication year to include papers published from all years up to 2018, (2) to search for English articles only, and (3) to use constraints while searching the digital libraries.

The search for the identified keywords on the identified databases above was conducted on November 15, 2018, and an additional search on PubMed was conducted on November 15, 2019, which returned a total of 2518 English articles that were published from all years up to 2018. The details of each database search are as follows:

1. Scopus yielded 627 English articles that were published from 1991 to 2018.
2. Web of Science yielded 378 English articles that were published from all years up to 2018.
3. ACM yielded 11 English articles that were published from all years up to 2018.
4. PubMed yielded 1502 English articles that were published from all years up to 2018.

**Table 1:** The result of testing the keywords for our article search.

Database	Search fields	Result with no constraints, n	Constraints	Result with constraints, n
Scopus	Title, abstract, and keywords	822	English, published in 2008 or after, and article	358
Web of Science	Topic	681	English, published in 2008 or after, and article	470
PubMed	MeSH^a^ fields	4627	English, published in 2008 or after, article, clinical trial, and human subjects	1502
ACM^b^	All fields	10	English, published in 2008 or after, and article	7
Total^c^	All fields	6140	All constraints	2337

^a^MeSH: Medical Subject Headings.

^b^ACM: Association for Computing Machinery.

^c^These results were affected by searching the libraries without constraints and yielded unrelated results.

### Step 2. Screening: Title and Abstract Review and Removing Duplicate References

After collecting 2518 articles, 200 duplicate articles were removed, which left 2318 articles for the screening process. The screening process was completed by all authors assessing the eligibility of each article until consensus was reached. After the screening review of each article’s title and abstract, 2049 articles were removed because they did not meet the inclusion criteria, which left 269 articles for the next step.

### Step 3. Eligibility: Inclusion and Exclusion Criteria

#### Overview

**Figure 1:**
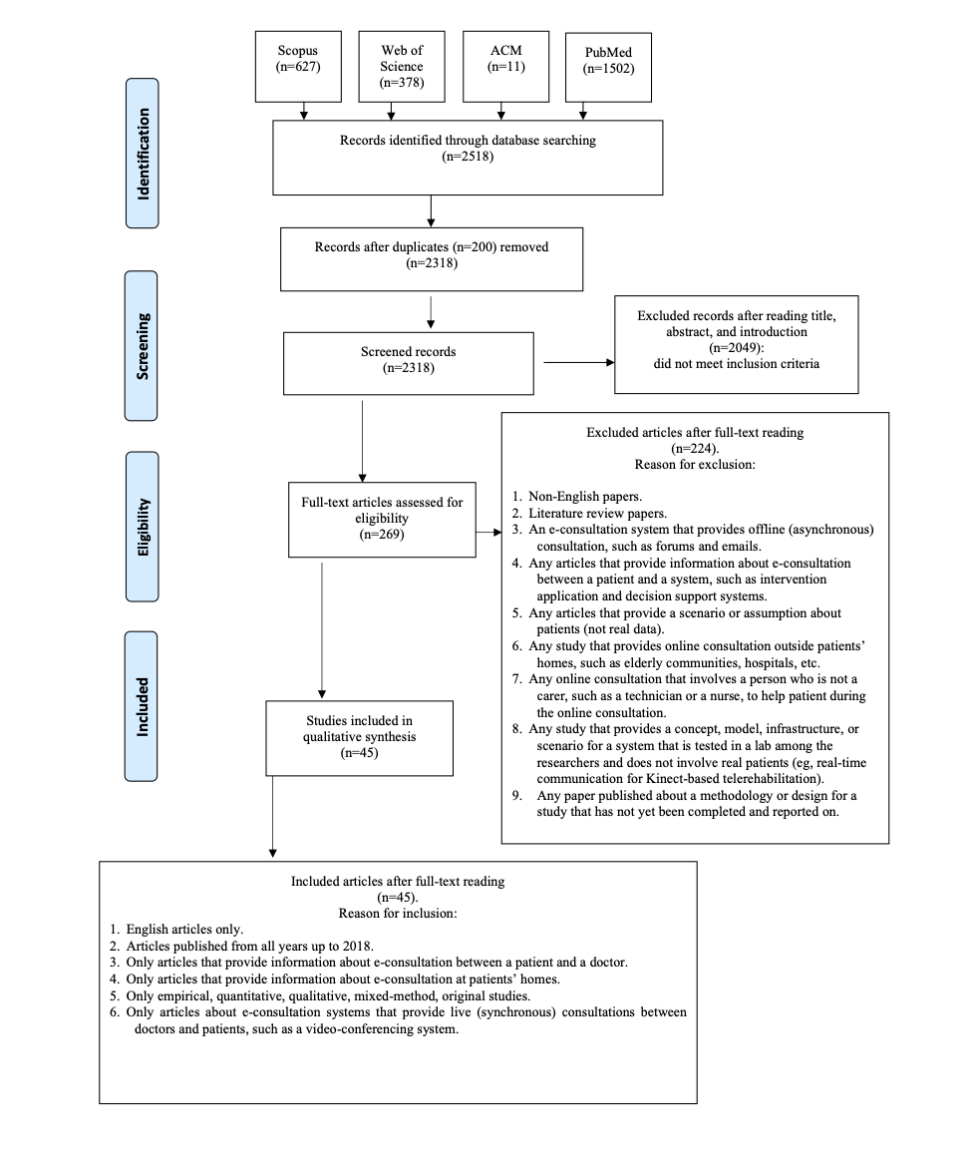
Summary of steps included in the article selection process displayed in a Preferred Reporting Items for Systematic Reviews and Meta-Analyses (PRISMA) flowchart. ACM: Association for Computing Machinery.

In this process, the inclusion and exclusion criteria were applied to the remaining 269 articles while conducting the full reading process (ie, the inclusion process). Figure 1 provides a summary of the inclusion and exclusion criteria and the steps of our search, which are illustrated in the Preferred Reporting Items for Systematic Reviews and Meta-Analyses (PRISMA) flowchart [18].

#### Inclusion Criteria

This research included the following: (1) original studies published in English, (2) studies published from all years up to 2018, (3) studies about any type of online health consultation between patients and health professionals, through any type of care provider, (4) studies about consultations performed remotely at patients’ homes (ie, e-consultation), (5) empirical, quantitative, qualitative, mixed-method, original studies, and (6) studies about e-consultation systems that provide live (ie, synchronous) video-conferencing systems.

#### Exclusion Criteria

This research excluded the following: (1) non-English articles, (2) literature reviews, (3) any articles about online consultation systems that provide offline (ie, asynchronous) consultations, such as forums and emails, (4) articles that provide information about e-consultation between a patient and a system, such as intervention applications and decision-support systems, (5) articles that provide a scenario or assumption about patients (ie, not real data), (6) articles about online consultations outside the patients’ homes, (7) articles involving a person who is not a patient carer, (8) studies that provide a concept, model, infrastructure, or scenario for a system that is tested in a lab among researchers without involving patients, and (9) papers that published their methodology or design for studies that have not yet been completed and reported on.

### Step 4. Inclusion: The Number of Included Articles for the Review

In this process, all authors assessed the 269 articles following the inclusion and exclusion criteria, and meetings were held discussing the eligibility of articles until consensus was reached. Thus, the authors included 45 articles in the final review, as shown in Figure 1.

### Data Extraction and Analysis

Manual qualitative content analysis was performed on the included studies in order to identify facilitators and barriers to real-time HOHC systems and the results were categorized into two main categories: facilitators and barriers [19]. In order to perform this thematic data extraction, the researchers predefined the facilitators and barriers to HOHC systems based on the Theory of Planned Behavior (TPB) and the Health Belief Model (HBM). According to the TPB, an individual’s intention to perform a certain behavior is influenced by internal and external factors. Internal factors are the individual’s characteristics, differences, knowledge, skills and abilities, emotions, and compulsions that influence the performance of intended behavior [20]. External factors are situational factors, such as time and opportunities, and depends on the action of other people who influence an individual’s control over the intended behavior [20]. According to the HBM, an individual will likely engage in health-related behaviors based on his or her perception of several variables that influence the uptake of health services. First, perceived susceptibility refers to the individual’s assessment of the possibility of getting a disease. Second, perceived severity is about the individual’s judgment of the seriousness of the disease. Third, perceived benefit reflects the individual’s evaluation of the effectiveness of the available action to reduce the threat of illness or disease. Fourth, perceived barrier refers to obstacles that prevent an individual from performing a healthy activity, such as high cost, time-consumption, side effects, and inconvenience. Fifth, cue to action refers to the internal and external process of decision making to perform or accept a healthy action. Finally, self-efficacy refers to an individual’s confidence in his or her ability to successfully perform a recommended health action [21,22].

Based on the above predefinition, facilitators to HOHC systems are the information and data gathered from examining positive feedback, comments, factors, and indicators mentioned in each article that helped in the users’ uptake of the system. Further, facilitators are divided into two subcategories: internal and external. The internal facilitators refer to the positive feedback, comments, factors, and indicators that have an effect on the user’s behavior and motivation while using the system. The external facilitators refer to the positive feedback, comments, factors, and indicators about the environment surrounding the system’s usage and the system itself.

The barriers to HOHC systems are the information and data gathered from examining negative feedback, comments, factors, and indicators mentioned in each article that hindered users’ uptake of the system. Further, the barriers are divided into two subcategories: internal and external. The internal barriers refer to the negative feedback, comments, factors, and indicators that have an effect on the user’s behavior and motivation while using the system. The external barriers refer to the negative feedback, comments, factors, and indicators about the environment surrounding the system’s usage and the system itself.

The first author (HKYA) analyzed all the articles and extracted the facilitators and the barriers to the HOHC system. The second and third authors (KTW and EVG) validated the results of the extracted information by performing a full-text reading of the articles. After that, several group meetings were held to discuss the discovered themes and data until consensus was achieved among all authors. The authors also extracted basic characteristics from each study, summarized their aims, and described the HOHC system that was used in each study. Further, they summarized the effectiveness of each HOHC system used in each article and the patients’ satisfaction with it.

## Results

### Overview

We analyzed each study qualitatively to extract themes of facilitators and barriers to HOHC systems. Also, the results of the analyses indicate that HOHC systems use video conferencing via different platforms as a medium to facilitate online consultations. Further, HOHC systems have been used for different types of diseases with patients of different ages and characteristics.

### Characteristics of the Studies

HOHC was provided to male and female patients with ages ranging from less than 1 year old to over 80 years old. It was also provided to patients with different health conditions and diseases (see Table 2). The duration of HOHC usage ranged from 2 weeks to 12 months and the studies were conducted in 11 different countries: United States (n=23), Australia (n=5), Canada (n=4), Italy (n=4), United Kingdom (n=2), China (n=1), Spain (n=1), Korea (n=1), Norway (n=1), Denmark (n=1), and Iran (n=1). Table 3 provides a comprehensive summary of the characteristics of each study [14-17,23-63]. Further, for detailed information about each study aim and the system used, see Table A1-1 in Multimedia Appendix 1.

**Table 2:** Health conditions and diseases addressed in the included studies.

Health condition, disease, or treatment	Count, n
Behavioral therapy	3
Burn injuries	2
Cancer	1
Cardiovascular disease	10
Chronic obstructive pulmonary disease	7
Cognitive rehabilitation	1
Diabetes	3
Facioscapulohumeral muscular dystrophy	1
Geriatric rehabilitation	1
HIV	1
Huntington disease	2
Multiple sclerosis	1
Parkinson disease	2
Peritoneal dialysis	1
Physical activity	2
Plastic surgery	1
Prader-Willi syndrome	1
Psychotherapy	4
Rehabilitation services for the elderly	1
Schizophrenia	1
Serious illness (not defined in the included articles, but an example was given, ie, cancer)	3
Stutter and speech therapy	2
Wound care	3

**Table 3:** Summary of the characteristics of the included studies.

Author	Location	Method	Sample size	Age in years	Health condition and/or treatment	Duration
Abdolahi et al [23]	Las Vegas, NV, USA	Cohort study (longitudinal) Nonprobability sample Clinical trial, baseline, and follow-up Montreal Cognitive Assessment	N=17	Mean 65.1 (PD^a^) Mean 57.7 (HD^b^)	PD (n=8) HD (n=9)	7 months (PD) 3 months (HD)
Armfield et al [24]	Brisbane, Australia	Cohort study (longitudinal descriptive)	N=92 (children at a regional hospital) N=2 (children at home)	No data	Clown Care for children with a serious illness or undergoing painful or distressing procedures	12 months at the hospital 8 months at home
Azar et al [25]	Northern California, USA	Cohort study (longitudinal) Randomized controlled trial Multiple measures Overall health-related quality of life measure at baseline and at 3 and 6 months postbaseline Physical activity measure Participant satisfaction measure	N=74 (adults) n=37 (in the delayed group: served as the control group) n=37 (immediate group)	Mean 59.7	Obesity Metabolic syndrome Prediabetes Type 2 diabetes Cardiovascular disease	Patients were assessed at baseline and at 3 and 6 months postintervention
Beck et al [17]	United States	Cohort study (longitudinal) Randomized controlled trial	N=927 (total) n=200 (eligible) n=195 (randomized) n=15 (dropout)	Mean 66	PD	12 months
Benton et al [26]	Florida, USA	Comparative study: nonrandom allocation of participants	N=1169 (treatment-as-usual) N=104 (TAO^c^ intervention) n=97 (out of 104: received intervention) n=72 (final sample: college students); 17 males and 52 females	Mean 21.72	Psychotherapy (anxiety)	7 weeks
Bernocchi et al [27]	Italy	Cohort design Patients were observed over 3 months and answered satisfaction questionnaire at the end of the study	N=15 (subacute group: stroke for less than a year) N=11 (chronic group: stroke for more than a year)	Mean 70 (SD 10)	Poststroke rehabilitation	3 months
Bull et al [28]	USA	Controlled trial Participant assessment at baseline and three random assessments in 4 months In the third month, participants completed the satisfaction survey	N=13 (participants who were randomized to receive three remote visits from one of two physicians)	Mean 56.5 (SD 16.6)	Motor assessment for patients with HD Virtual visits in HD	4 months
Burkow et al [29]	Northern Norway	Mixed-method pilot study Participants were assessed at baseline, assessed, and interviewed after the study (semistructured interview and questionnaire)	N=10	Mean 61.7	COPD^d^ rehabilitation	9 weeks
Choi and Kim [30]	Seoul, South Korea	Controlled trial: a nonequivalent control group, pre- and posttest, quasi-experimental study	N=25 (experimental group: blood pressure monitoring and video consultation twice a week) N=24 (control group: only blood pressure monitoring)	>65 (patients)	Hypertension (blood pressure)	8 weeks: 16 sessions conducted twice a week
Demiris et al [31]	University of Minnesota, USA	﻿Experimental study: randomized controlled trial with a survey (measured perceptions of telehome care before and after the intervention)	N=17 (experimental group: 9 male and 8 female patients) N=11 (control group: 5 male and 6 female patients)	Mean 76.75 (experimental group) Mean 75.55 (control group)	COPD Congestive heart failure Those requiring wound care	No data
Dimitropoulos et al [32]	Cleveland, OH, USA	Cohort study: evaluation of pre- and postintervention assessment for children Survey for parents (online modified version of Behavioral Intervention Rating)	N=10 (total: 7 males and 3 females) n=8 (final)	6-12	Prader-Willi syndrome	6 weeks (12 sessions)
Edwards and Patel [33]	Maine, USA	Comprehensive retrospective review Data collected from different stages of system development (network data and questionnaire about health providers’ and patients’ satisfaction and system effectiveness)	N=86 (patients)	Not reported	Chronic heart disease (42%), cancer (23%), and lung disease (14%) Almost 20% had diabetes as a secondary diagnosis	3 years
Ehlers et al [34]	United States	Observational study: randomized controlled study (two arms) Mixed method of data collection Satisfaction survey Interviews System evaluation and quantitative outcomes assessment	N=30 (total) n=15 (control group) n=15 (tablet group)	30-64 (middle-aged women)	Intervention to improve physical activity	12 weeks
Eslami Jahromi and Ahmadian [35]	Iran	Descriptive analytical study Researcher-made questionnaire administered after the session and after 2 weeks follow-up	N=30 (56.7% male and 43.3% female patients)	14-39	Advanced stutter	3 months
Finkelstein et al [36]	Minnesota, USA	Randomized and controlled trial: patients were randomly assigned to one of three groups Multi-measures Mortality and morbidity; transfer to a different level of care and cost	N=68 (patients) n=53 (completed the study)	Mean 74.3	Patients with asthmatic or chronic disease Spirometry for asthma or COPD Wound dressing procedures for diabetic patients	6 months
Finkelstein et al [37]	Minnesota, USA	Randomized controlled trial: patients were randomly assigned to one of three groups Participants completed a questionnaire to measure their perceptions, satisfaction, and usefulness of TeleHomeCare at the start and end of 1 month of the intervention	N=68 (patients) n=53 (completed the study)	Mean 74.3	Patients with asthmatic or chronic disease Spirometry for asthma or COPD Wound dressing procedures for diabetic patients	6 months
Garcia et al [38]	United States	Cross-sectional retrospective study comparing patients who received standard assessment and treatment with patients who received assessment and treatment over the burn app	N=67 (total) n=35 (TeleBurn app treatment) n=32 (face-to-face treatment)	Mean 4.9	Burn injuries	9 months
Ghio et al [39]	Italy	Cohort study (nested case-control study)	N=2 (patients)	12 (girl) 10 (boy)	Peritoneal dialysis	7 months
Green et al [40]	United States	Randomized controlled trial Baseline, postintervention, and 6-month follow-up assessments using semistructured telephone interviews	N=396 (total) n=71 (randomized) n=36 (intervention group) n=35 (wait-list crossover group)	Mean 44	Women living with HIV	Follow-up after 6 months
Guillén et al [41]	Spain	Cohort study (longitudinal) Three different groups were given questionnaires to assess participants’ feelings and acceptance of global usability of the system	N=50 (group A: gynecology patients tried the system at the doctor’s office) N=2 (group B: pregnant women used the system from their homes) N=10 (group C: students at the medical center)	Not reported	Chronic ill people, elderly people, and any person who may require health attention at home	3 months (group B)
Harris et al [42]	United States	Cohort study (longitudinal) Controlled randomized clinical trials Before, after, and follow-up assessments of participants’ adherence and glycemic control	N=138 (total) n=90 (final: youth) n=46 (Skype group) n=44 (clinic group)	Mean 15.04	Behavioral Family Systems Therapy for Diabetes (BFST-D)	12 weeks
Hickey et al [16]	Massachusetts, USA	Retrospective cohort study (longitudinal) Multiple data were collected for the system and for system usage analysis and evaluation	N=31 (27 males and 4 females)	Mean 44	Burn care	15 months
Hwang et al [43]	Australia	Cohort study (longitudinal) Randomized controlled trial Mixed-methods design with purposive sampling (self-report surveys and semistructured interviews)	N=17	Mean 69	Rehabilitation for heart failure patients	12 weeks
Kasschau et al [44]	United States	Placebo-controlled study (open-label study) Participants reported before-and-after *brief adverse event reports* and completed self-report measures of treatment tolerability	N=20	Not reported	Multiple sclerosis	2 weeks
Mariano et al [45]	United States	A longitudinal design (participants served as their own control group) Measurements were done at baseline, pretreatment, and posttreatment for cognitive skills and behavior function assessments	N=21	Mean 14.61	Schizophrenia: cognitive remediation for adolescents with 22q11 deletion syndrome (22q11DS)	8 months (no intervention) 8 months (cognitive remediation intervention)
Marziali and Donahue [46]	Canada	A randomized controlled study Participants completed health status and stress-response measures at baseline and at 6-month follow-up	N=66 (total: family caregiver assigned to three forums) n=22 (in each forum: Alzheimer disease, stroke-related dementia, and PD)	Mean 67.8	Psychosocial and educational intervention for family caregivers of older adults with neurodegenerative disease	10 weeks (intervention: follow-up after 6 months)
McCrossan et al [47]	United Kingdom	Cohort study (longitudinal) Randomized controlled trial employed qualitative analysis of participants using structured questionnaires	N=83 (total) Participants were allocated to three groups randomly: two intervention and one control n=35 (video conferencing) n=24 (telephone) n=24 (control)	<1 (infants)	Babies with congenital heart disease	41 months
Melton et al [48]	Colorado, USA	Cohort study (longitudinal) Participants completed online questionnaire to provide quantitative and qualitative feedback and evaluation of the system	N=8	18-40	Mental health support for patients with cancer	6 weeks
Peel et al [49]	University of Queensland, Australia	Experimental study (longitudinal) Patients were prospectively recruited to the trial Staff completed satisfaction survey and provided qualitative feedback about the system and patients’ usage of it	N=44 (patients)	Mean >80	Geriatric rehabilitation	8 months (data collection)
Pietrabissa et al [50]	Italy	Three-phase cross-sectional survey study Questionnaire at different stages: baseline, after consultation, and follow-up after 1 month	N=284	Mean 29.9	Consulting psychology	Baseline survey before the consultation, second survey after the consultation, and third survey after 1-month follow-up
Portaro et al [51]	Italy	Evaluation study (case control) Multiple surveys used at baseline and at the end of the study to measure participants’ psychological aspects	N=4 (siblings)	Not reported	Facioscapulohumeral muscular dystrophy	6 months
Rosen et al [52]	United States	Cohort study (longitudinal) Compare 6-month intervention with prior 6-month control period of the same patients	N=50 (patients)	Mean 61	Congestive heart failure	6 months
Tam et al [53]	China	Cohort study (single case and qualitative research design) Multiple memory test measures and interview to assess participants before and after the rehabilitation and questionnaires to measure patients’ satisfaction	N=3	37 (case 1) 20 (case 2) 20 (case 3)	Cognitive rehabilitation for functional performance	All patients received six training sessions; no data on the duration
Taylor et al [54]	Flinders University, Australia	Action research process (quantitative and qualitative data collection) Questionnaire to assess users’ experience of the system and multiple tools to assess the system performance	Not reported	>65	Rehabilitation services for the elderly	Not reported
Thomas et al [55]	University of Sydney, Australia	Quasi-experimental study (multiple baseline design) Multiple measures for children’s abilities and skills and parents’ satisfaction: semistructured, clinical feedback	N=5	5-11	Rapid Syllable Transitions (ReST) treatment for children with childhood apraxia of speech	3 weeks (treatment)
Vijayaraghavan et al [56]	Newham, UK	Observational study (cross-sectional survey and interview) Mixed-method, quantitative, online questionnaires and qualitative interviews with patients (15 face-to-face, 19 in-depth, and 5 focus groups)	Not reported	﻿<50-79 (62% of those who agreed to participate)	﻿Diabetes appointments via webcam	Not reported
Vismara et al [15]	United States	Experimental study (a single-subject, multiple-baseline design) Participants completed three measurements at baseline, during the intervention, and at 3-month follow-up	N=8 (families: 7 mothers and 1 father)	<4	Intervention for parents to improve their skills in improving behaviors of children with autism	12 weeks
Walsh and Coleman [57]	Connecticut, USA	Observational descriptive study Case study on 2 patients with chronic disease	N=12 (total) n=2 (in this study)	82 (female) 77 (male)	Chronic disease: diabetes and heart disease	Not reported
Westra and Niessen [58]	Netherlands	Cohort study (longitudinal) Randomized controlled trial employed an online survey using validated measurements to assess patients’ satisfaction, communication experiences, and time spent on consultations	N=52 (total patients) n=46 (included in the study) n=25 (online consultation) n=21 (in-person consultation)	>18	Follow-up for patients who underwent plastic surgery	6 months
Williams et al [59]	Massachusetts, USA	Cross-sectional study Online screening survey using Patient Health Questionnaire (PHQ-9), participants’ feedback survey after 1 week of the online consultation, and follow-up survey (PHQ-9) after 8 weeks	N=972 (total) n=285 (screened positive for depression) n=17 (successfully completed the Skype consultation)	Not reported	Depression	One-time Web-based consultation
Woodend et al [60]	Canada	Cohort study (longitudinal) Randomized controlled trial with random allocation to intervention	N=249 (total patients with symptomatic heart failure and angina) n=121 (heart failure) n=128 (angina)	Mean 66	Cardiac diseases: heart failure and angina	Data were collected at three stages of using the system: 1 month, 3 months, and 1-year postdischarge
Wu and Keyes [14]	Burlington, VT, USA	Cohort study (longitudinal) Exit interview questionnaire to measure (1) participants’ satisfaction and acceptance of the program, (2) exercise effectiveness, and (3) participants' compliance with the exercise	N=17 (elderly: 13 females and 4 males)	Mean 81	Improving balance in elders	15 weeks
Young et al [61]	Canada	Cross-sectional study (descriptive) Qualitative method Three semistructured interviews were used: prestudy, during the study, and poststudy	N=63 (total families) n=16 (families included in the study)	<1	Life-threating health conditions	6 weeks
Young et al [62]	Canada	Cohort study (comparative analysis) Nonrandomized controlled trial Measurement tools for both children and parent: Quality of Life scale used before and after discharge; Impact on Family (IoF) scale used at baseline, 1 week, 2 weeks, and 8 weeks	N=63 (total) n=50 (patients who participated in this study)	<5	Multisystem disorders: cardiac; respiratory; and ear, nose, and throat diseases	6 weeks
Sorknaes et al [63]	Denmark	Cohort study (longitudinal) Randomized controlled trial Main-measures outcome: comparing hospital readmissions between intervention and control groups	N=266 (total patients) n=132 (intervention) n=134 (control)	Mean 72	Acute exacerbation COPD	26 weeks (total study; 1 week of real-time consultation)

^a^PD: Parkinson disease.

^b^HD: Huntington disease.

^c^TAO: Therapist-Assisted Online.

^d^COPD: chronic obstructive pulmonary disease.

### Home Online Health Consultation Systems

HOHC systems in all reviewed articles featured the use of synchronous video conferencing systems or software as a medium to facilitate the communication between a health professional and a patient or a patient’s carer. The video conferencing feature was a part of a complex telemedicine system or a simple stand-alone software program on a patient’s mobile phone or personal computer. The results showed that 25 of the studies conducted online consultation via specially developed telemedicine systems that provide video conferencing as part of its main services. The remaining studies used off-the-shelf video conferencing software to conduct the home online consultation. In total, 4 studies used Skype software, 4 studies used Vidyo software, 5 studies used Web-based video conferencing systems, 2 studies used Adobe Connect, and other studies used different platforms, including Cisco WebEx, Moodle, Cisco Jabber, Facebook Messenger, or the Microsoft NetMeeting system (see Table A2-1 in Multimedia Appendix 2).

The complexity of the HOHC system used was related to the complexity of the patient’s health condition. If a patient had multiple and complex health conditions, a complex telemedicine system was used for monitoring his or her health condition. In contrast, when a patient had a single health condition, a simple system was used for remote treatment (see Table 4).

**Table 4:** Complexity of Home Online Health Consultation (HOHC) systems.

System	Use of the system
Skype	Patients who had stuttering issues [35] Families that include patients with diabetes for behavioral therapy [42] Online screening of patients with depression [59]
Vidyo	Rehabilitation services [54] Burn care [16] Motor assessment of patients with Huntington disease [28] Provide remote care for patients with Parkinson disease [17]
Adobe Connect	Childhood apraxia of speech [55] Rehabilitation for patients with heart failure [43]
Cisco WebEx Web conferencing	Cognitive remediation for adolescents with 22q11 deletion syndrome [45]
Microsoft NetMeeting	Cognitive rehabilitation to improve the functional performance of patients who had a brain injury [53]
Facebook	Provide remote psychology consultation [50]
Cisco Jabber	Patients who underwent plastic surgery [58]
Web-based video conferencing	Rehabilitation for patients with chronic obstructive pulmonary disease [29] Intervention for parents to improve their parenting skills and help to improve their children’s ability to communicate [15] Psychosocial and educational therapy for family carers of older adults with neurodegenerative diseases [46] Mental health support for patients with cancer [48] Online assessment of patients with movement disorders [23]
Moodle	Anxiety intervention with college students [26]

### Home Online Health Consultation Effectiveness: Video Conferencing

The effectiveness of an HOHC system to deliver remote consultation is determined by its ability to achieve the health care outcomes as reported by the authors of each article. Out of 45 included studies, 44 (98%) reported that online consultation systems were effective in improving patients’ overall health conditions and in assessing patients’ health conditions successfully. However, the level of evidence [64] is different in each study, ranging from Level II to Level VI. Despite this different in range, the included studies presented a medium-high strength on the level-of-evidence grade [64], with the majority of the articles falling under Level II and Level IV (see Table A3-1 in Multimedia Appendix 3).

Several studies reported that the online consultation was effective and was as good as in-person consultation [14,27,28,32-35,37,38,46,54,55]. However, 1 study reported that patients preferred a combination of online consultation and face-to-face consultation [43], and 2 studies reported that participants preferred face-to-face consultation [34,56].

On the other hand, the study by Peel et al [49] reported failure in implementing a home telerehabilitation program of geriatric rehabilitation for elderly people. The program faced challenges with patients who had low mobility, complex social problems, low hearing and vision, and cognitive impairment. Also, patients required assistance from a third person to use the system. However, the authors concluded that the system had the potential to deliver remote rehabilitation services, but it faced many barriers that needed to be overcome to ensure its effectiveness.

### Participants’ Satisfaction With Home Online Health Consultation

In total, 12 studies reported on participants’ satisfaction with the use of HOHC systems. In Thomas et al [55], parents’ satisfaction with the remote treatment received an average score of 9.5 out of 10. In the study of Eslami Jahromi and Ahmadian [35], 16 out of 30 patients were satisfied with the teletherapy services. Bernocchi et al [27] reported that 100% of patients were satisfied with the program: 60% were very satisfied and 40% were satisfied. Dimitropoulos et al [32] reported that participants rated their satisfaction with the program with a mean rating of 4.71 out of 5. In Azar et al [25], the overall satisfaction with the program was high, with a mean rating of 4.1-4.4 out of 5. All patients in the Woodend et al [60] study showed high satisfaction with the program at different stages: 92% in the first month, 92% in the second month, and 97% in the third month. Walsh and Coleman [57] found that patients’ satisfaction with the program was overwhelmingly favorable. In the Pietrabissa et al [50] study, the level of participants’ satisfaction was rated as 4 out of 5. Further, Edwards and Patel [33] reported that 95% of patients and 98% of health care staff were *very satisfied* with the 2619 home televisit sessions. In Westra and Niessen [58], patients’ satisfaction with the HOHC system received a mean rating score of 3.91 out of 5. Also, in the Portaro et al [51] study, participants reported that the system was reasonable and user friendly. In the study by Wu and Keyes [14], all 17 participants expressed a favorable opinion of the program.

### Facilitators and Barriers to Home Online Health Consultation

The identified facilitators and barriers from each article are summarized in Table 5 (facilitators) and Table 6 (barriers), which provide results for each facilitator and barrier addressed in the included studies. Further, thematic extraction of facilitators and barriers from each article can be found in Table A4-1 in Multimedia Appendix 4.

HOHC systems in all reviewed articles required patients to have access to the Internet and phone line services to receive the needed health care services at their home. All studies used Internet access as an inclusion criterion in order to participate in the study. However, some studies reported that participants dropped out due to later Internet connection issues [14,45,55,56].

Internet speed that affected the quality of the HOHC was mentioned in 20 studies. In total, 15 out of 20 studies (75%) reported that slow Internet speed during the consultation resulted in poor video and audio quality, loss of connection, and participants’ frustration [14,23,27,28,32,33,35,39,40,42,43,47, 53-55]. On the other hand, fast Internet speed was reported in 5 out of the 20 studies (25%), which had a positive impact on the communication quality between patients and care providers [17,28,42,54,55].

Poor signal from the wireless and 3G networks was reported in 3 studies, which affected the quality of the online consultation [29,42]. For example, the wireless and 3G network signals were affected by the home interior and the weather conditions [54], which reduced the wireless and 3G signal strength.

Ease of use of the HOHC system was related to how easily patients and clinicians were able to navigate and use its services. Ease of use was reported by patients and clinicians in 22 studies as a key factor of system effectiveness, high satisfaction, and the acceptability of the HOHC system [16,17,24,25,28-30, 32,35,37,40-43,48,49,51,53,55,57,61]. On the other hand, in 7 studies some participants reported that they had difficulty using the online consultation system. Most of them reported that they had difficulty navigating or installing the system on their computer [14,15,34,39,53,58,60].

Participants’ familiarity with the technology used for HOHC was reported in 6 studies and helped them to accept and adapt to it faster [26,29,35,41,56,59]. For example, participants were familiar with Skype because it is a popular platform for online communication [35]. In contrast, patients’ lack of knowledge, unfamiliarity with communication technology, and fear of the unknown resulted in resistance to the use of the HOHC technology, which was reported in 11 studies [23,26,33,34,36,37,41,43,54,56]. Also, nurses’ resistance to the system limited the uptake of the HOHC technology [57].

Patients’ training was reported in 20 studies, which helped patients to use the system and its equipment easily [16,24,27,30,31,35-37,39,40,46,47,49,53-55,57,62]. Training was provided by the health care provider to patients before starting the online therapy; training was given in the following forms: face-to-face [25,30,39,61,62], through video orientation, or though manual documentation [32]. The type of training provided depended on the type of health condition and the specific online consultation system. For example, participants in the multiple sclerosis study completed 192 online sessions, 40 of which were online training sessions [44]. However, the lack of patients’ training and knowledge regarding technology use was considered as a barrier to HOHC in 3 studies [23,30,41].

Training for clinicians to use the online consultation system was reported in 10 studies. In-person training aimed to familiarize clinicians with the system, the system’s equipment, and the treatment procedures [15,25,26,30-32,36,39,46,49,52, 55,56]. In addition, few authors reported that individual clinicians’ skills, such as communication skills, helped in delivering the best care for patients and contributed to the treatment plan in some studies [24,25,45,63]. However, lack of staff training affected the uptake of online consultation. For example, Peel et al [49] indicated frequent changes of staff during the study and their lack of training limited the uptake of the eHAB™ system.

Saving costs was reported in 21 studies as an advantage of using HOHC. In some studies, cost savings were calculated based on the cost of the traveled mileage per patient [16] or were reported without details of cost savings [15,23,31,35,38,42-44, 48,50,56,58]. Other studies compared the cost of online consultation to traditional face-to-face consultation [14,26,29,33,36,39,47,57].

Reducing travel time was reported in 15 articles as an advantage of using HOHC. Participants reported that online consultation eliminated the burden of traveling from home to health center or outpatient unit [15,16,23,28,29,31,35,42-44,48,54,58]. In addition, both reducing travel and waiting times were reported in 9 articles as an advantage of using HOHC. Patients reported that HOHC eliminated their waiting times for therapy [15,23,28,31,35,38,44,58] and clinicians reported that it reduced their travel time; thus, there was no waiting time [54].

**Table 5:** Facilitators of Home Online Health Consultation found in articles.

Facilitators	References of the studies where facilitators were addressed or discovered	Total count, n
Saving costs	[14-17,23,24,26,30,36,38,41,42,45,47,48,50-52,54,57,61]	21
Reducing waiting time	[15,23,28,31,35,38,44,54,58]	9
Reducing travel time	[15,16,23,28,29,31,35,42-44,48,54,58]	15
Easy to use	[16,17,24,25,28-30,32,35,37,40-43,48,49,51,53,55,57,61]	22
Familiarity with the system	[26,29,35,41,56,59]	6
Trust in technology	[48]	1
Involvement of family	[15,27,32,39,43,51,62]	7
Patients’ ages	[32]	1
Patients’ training	[16,24,27,30,31,35-37,39,40,46,47,49,53-55,57,62]	20
Clinicians’ training and skills	[15,25,26,30-32,36,39,46,49,52,55,56] and [24,25,45,63]	13 and 4
Patients’ familiarity with staff and past experience	[27,29,31] and [50]	3 and 1
Convenience	[14,15,17,31,40,42,43,48,50,55,59]	11
Motivation and engagement	[14,15,26,32,46,50,53,55]	8
Providing support: emotional, technical, and organizational	[15,26,29,43], [14,16,44,53,58], and [33,57]	4, 5, and 2
Enabled body language	[41,59]	2
Fast Internet speed	[17,28,42,54,55]	3
Internet or phone availability	[17,23-28,33,34,36,47,55,57,60,61]	15
Insurance coverage	[45]	1
Security	[16,26,35-38,46,51,58,59]	10
Privacy	[14,23,26,30,31,36,37,41,48,53,57,61]	12
Better management	[15,37,38,43,44,48,53]	7
System approach to improve patients’ compliance	[26,37,38,43,44]	5
Improved accessibility to care	[24,35,38,42,43,45,51,59,62]	9
Developed with expert	[38]	1

**Table 6:** Barriers to Home Online Health Consultation found in articles.

Barriers	References of the studies where barriers were addressed or discovered	Total count, n
Slow Internet speed	[14,23,27,28,32,33,35,39,40,42,43,47,53-55]	14
Poor audio quality	[14,16,23,28,32-34,43,55,57,61]	11
Poor video quality	[14,16,23,28,32,33,43,55,57,60,61]	11
Internet access issue	[14,45,55,56]	4
Poor signal coverage	[54]	1
Wireless issue or poor signal	[29,42,54]	3
Hard to express emotion (patients)	[34,35]	2
Lack of body language	[24,31,34,58]	5
Low physician communication skills	[58]	1
Resistance to technology	[23,26,33,34,36,37,41,43,54,56,57]	11
Patients prefer face-to-face	[30,50,56]	3
Patients’ lack of seriousness	[35]	1
Environmental obstruction	[32,34,40,49,53,59]	7
Patients’ health conditions	[48,49]	2
Patients required assistance	[49]	1
System still under development	[38]	1
Difficult to use the system	[14,15,34,39,53,58,60]	7
Difficult to place the camera	[14,28,32]	3
Technological incompatibility	[17]	1
Required restarting the computer	[16]	1
Lack of system cross-synchronization	[15,57]	2
Scheduling conflicts	[34,48,59]	3
System and device size and weight	[49]	1
Security concerns or issues	[40,42,59]	3
Privacy concerns or issues	[29,31,40,42,59]	5
Reimbursement issues	[16,17,26,29,33,36,41,61]	9
Policy and law issues	[17,26,36,41,61]	5
The system is expensive	[41]	1

Security was reported in 10 studies as an advantage of using HOHC to protect the patients’ information. Skype was used in a few studies, which provides 256-bit encryption as a security feature [35,59]. Further, other studies used Vidyo as a platform, which provides a higher level of security as it is compliant with the Health Insurance Portability and Accountability Act (HIPPAA) [16]. Some studies implemented security in their system to comply with HIPPAA [38], and other studies did not specify the type of implemented security features [26,36,37,46,51,58]. However, few studies indicated that participants had security concerns about online consultation [40,42]. Further, Skype is not HIPAA compliant, as mentioned in Williams et al [59], and may not provide a high level of security.

Privacy was reported in 12 studies in terms of saving participants’ data privately and creating a sense of privacy at home while conducting the online consultation [14,23,26,30,31,36,37,41,48,53,57,61]. In contrast, 5 studies indicated that participants had a concern about their privacy. Participants who had not disclosed their health condition to their family indicated that during the online consultation the information might be overheard by one of their family members [42,59]. Also, participants reported that someone living at home could be seen by others during the video conferencing consultation [29,31,40].

Managing participants over the HOHC was reported in 7 studies as an advantage. Clinicians reported that the online consultation system was faster at documenting and saving patients’ records [38]. Also, online consultation provided greater scheduling capability and flexibility for patients and clinicians [15,37,43,48,53]. Further, some methods of online consultation enabled clinicians to take control over participants’ computers when necessary [44]. However, other studies reported that patients had time and scheduling conflicts with the online treatment time [34,59] due to a medical appointment or medical emergency, which made them skip some of the online treatment sessions [48].

Participants in 11 studies reported that HOHC was convenient at meeting their health needs. The convenience aspect of the online consultation was related to eliminating travel and waiting times, saving costs, and completing the consultation from the comfort of their home at any time [14,15,17,31,40,42, 43,48,50,55,59]. On the other hand, in the Ehlers et al [34] study, some of the women reported that wearing an accelerometer was inconvenient. In Peel et al [49], the weight and size of the device made it challenging for patients to carry around the home and to store at home due to the lack of sufficient space.

Motivation and engagement were reported in 8 studies as advantages of conducting HOHC. Motivation and engagement were related to the encouraging and engaging communication between therapist and participants and among participants themselves [14,15,26,32,46,50,53,55]. However, 1 study reported that patients’ engagement was difficult with young children present [24].

The HOHC system approach enabled better treatment compliance for patients. It was reported in 5 studies that patients showed better compliance, adherence, and accountability to the overall treatment, which helped them to recover faster during online treatment [26,37,38,43,44].

HOHC improved patients’ access to health care services, which was reported in 9 studies. Patients reported that online consultation helped them to gain access to specialist care [42,51], to general care, and to real-time assessment [24,35,38,43,45,59,62]. However, 1 study reported that participants had less access to care providers, due to a technical issue. For example, in the Westra and Niessen [58] study, patients in the online group perceived accessibility and convenience to be lower, compared to the patients in the control group, due to lack of physical presence.

In total, 2 studies reported that online consultation enabled body language communication and created a feeling of presence between patients and therapists. For example, Williams et al [59] reported that online consultation enabled psychologists and psychiatrists to read patients’ body language cues. Participants also rated the feeling of presence with a score of 4.17 out of 5 during their online therapy session [41]. On the other hand, several studies reported that body language and social presence were disadvantages of online consultation. For example, lack of eye contact and emotional expression were reported by 24 participants, who said that it made them feel uncomfortable [35]. Further, 5 women reported that lacking a social presence made them feel disconnected during the online consultation [34]. Also, 52% of elderly patients, with a mean age of 76.75 years, reported that they did not like the lack of physical contact during a TeleHomeCare visit [31]. Moreover, clinicians reported difficulties engaging with very young children during the online therapy [24] or reported an inadequate ability to physically examine their patients [58].

Patients reported that their interactions with the clinical staff during face-to-face treatment before the online consultation helped them to be more comfortable and to have a good relationship with clinical staff during the online consultation [27,29,31]. Furthermore, patients’ past in-person treatment experience with a therapist encouraged them to participate in online consultation treatment [50].

Several studies reported that providing feedback and technical support helped in the uptake of online consultation. Participants reported that they felt well-supported during the online consultation by clinicians [15,26], which helped them to be more social with others [29,43]. The technical support helped participants overcome technical issues in a timely manner [14,16,44,53,58]. In total, 2 studies reported that organizational support [33] and health insurance coverage helped in the uptake of the online consultation [57]. However, lack of online consultation system integration and cross-synchronization with another system at the hospital prevented documentation of patients’ records [57] and prevented them from accessing desired features offered by other platforms [15]. Also, incompatibility of the HOHC technology with patients’ devices prevented them from participating in the online consultation. For example, 5 individuals withdrew from the Beck et al [17] study because the Vidyo software did not work with their old operating systems. Several studies indicated that law and policy prevented the uptake of online consultation. Most of the laws and policies were related to legal issues and reimbursement that the health organization did not support [16,17,26,29,33,36,41,61].

Involving health care experts during the development of the online consultation application enabled better patient experiences. For example, 1 study reported that the development of the TeleBurn app involved pediatric burn care, health communication, nursing, public health, biostatistics, information technology, and clinical psychology experts, which resulted in an app that helped patients to heal faster and to comply to the treatment better than face-to-face treatment [38].

Several studies reported that family involvement during the treatment to provide the care needed for their family member helped in the uptake of the online consultation. Family carers were involved in the treatment of family members who needed assistance from the beginning of the treatment, especially with patients younger than 12 years old or above 69 years old [15,27,32,39,43,51,62]. In contrast, 1 study reported a failure in implementing online consultation for the elderly because it required assistance from a third person and no family members were available for the study [49].

In total, 7 studies reported that home layout complications and lack of a dedicated room to conduct the online consultation reduced the quality of the consultation. Distractions from the surrounding home environment reduced patients’ attention during many online sessions because other family members were doing other home tasks, such as cooking, watching TV, answering the phone, and talking with other members of the family [32,34,40,49,53,59].

## Discussion

### Overview

In this article, we reviewed 45 studies that used HOHC systems to deliver real-time remote health care services to patients in their homes. This review contributes to the literature by conceptualizing a framework of facilitators and barriers to HOHC. In this section, we discuss the framework and then outline the practical implications and limitations of this review.

### Facilitators and Barriers to Home Online Health Consultation

The Results section identified a framework of four requirements—17 facilitators (10 internal and 7 external) and 8 barriers (5 external and 3 internal)—categorized as internal and external influencers on HOHC, as shown in Table 7.

**Figure 2:**
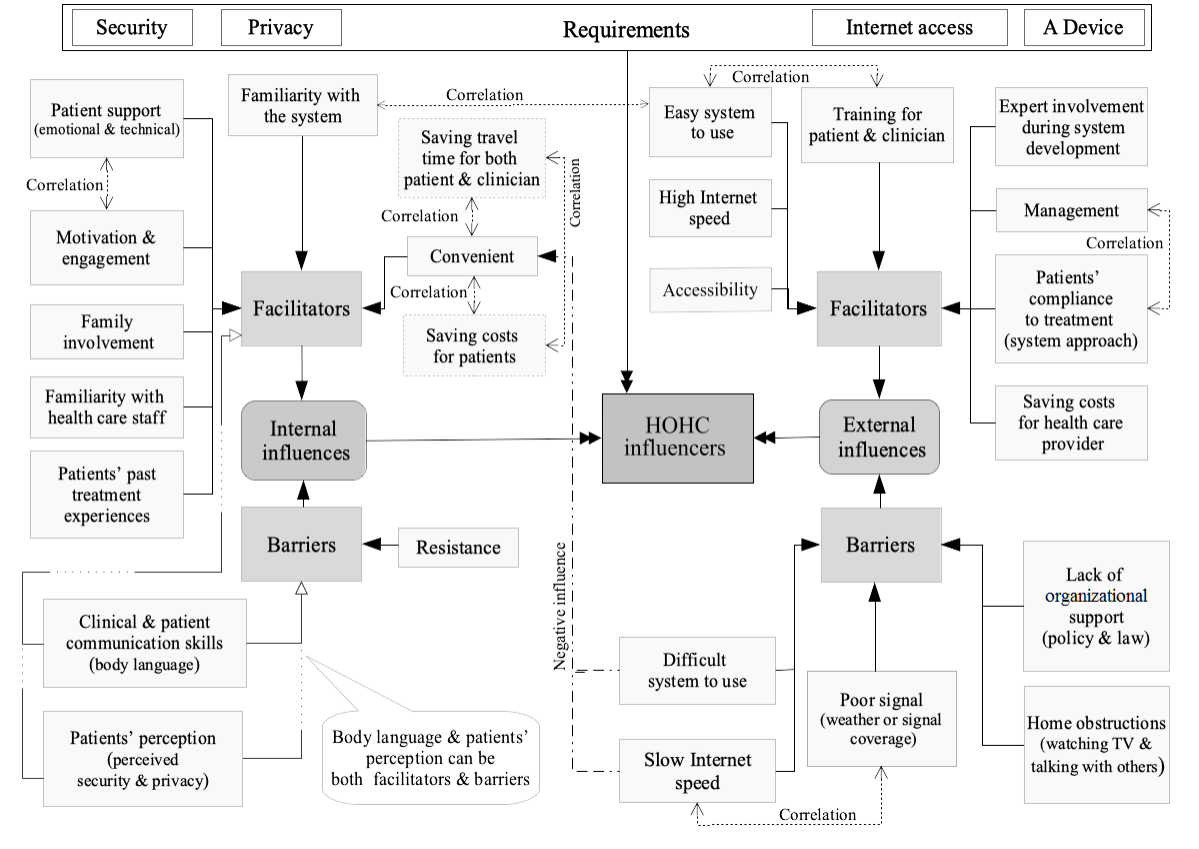
Illustration of internal and external influencers: facilitators and barriers. HOHC: Home Online Health Consultation.

External factors refer to the environment surrounding the system’s usage and the system itself that influence users’ acceptance and use of HOHC services [20-22]. This includes the technological capabilities of the HOHC system and the user capabilities of the patient and the clinician. The technological capabilities include the representation of the information, since online consultation simulates a face-to-face consultation between patient and health professional [64]. In fact, the results showed that patients and clinicians are able to communicate over video conferencing, and some patients are willing to pay for the online consultation because they found that it provides a similar experience to an in-person consultation. User capabilities include the way the patient explains or presents his or her health condition to his or her health professional, as well as the way the health professional interviews the patient to elicit all the needed information in order to perform a successful diagnosis [64]. Internal factors refer to the users’ behaviors and motivations while using and interacting with the system, which are keys to patients’ acceptance of the use of this technology. These factors include patients’ beliefs and patients’ perceptions of the relative advantages and disadvantages of HOHC compared with existing health care practices [20-22]. Figure 2 provides an illustrative overview of the identified framework of requirements, facilitators, and barriers to HOHC, as well as the correlation between facilitators and barriers.

**Table 7:** Internal and external facilitators and barriers.

Type of influencer^a^		Details
**Facilitator**		
	Internal	Time saved Convenience Familiarity with the system Patients’ past treatment experiences Patients’ familiarity with clinicians Family members’ involvement Engagement and motivation Excellent body language and communication Providing emotional and technical support to patients Patients’ positive perceptions of Home Online Health Consultation privacy and security
	External	High Internet speed Saving costs on health care services System ease of use Training for both patients and clinicians System’s approach to enforce patients’ compliance with treatment Management Accessibility
**Barrier**		
	Internal	Resistance Poor body language and communication Patients’ negative perceptions of Home Online Health Consultation privacy and security
	External	Slow Internet speed Poor network signal System difficult to use Lack of organizational support Home obstructions

^a^Types of influencers are listed, keeping in mind the requirements of a Home Online Health Consultation system: (1) security, (2) privacy, (3) Internet service availability, and (4) availability of a device.

### Home Online Health Consultation Requirements

There are four requirements for HOHC. *Security* and *privacy* are very important requirements because the communication supported by HOHC is personal and confidential. The security and privacy of the HOHC can be considered from the aspect of its compliance with the HIPAA. This act sets the standard for security and privacy for patients’ sensitive health information and records that are held or transferred in electronic form between health care providers and patients [16,28]. Another requirement is the *Internet service* *availability* for this type of consultation, without which patients cannot access online consultation. The *availability of a device* is a requirement and it can be either a personal device (eg, mobile phone, tablet, or PC) or a telemedicine device provided by the health care provider to patients. These requirements are essential for delivering any HOHC, and online consultation cannot be performed without them.

### External Facilitators and Barriers (Influencers) to Home Online Health Consultation

High Internet speed affects the quality of the consultation and can positively influence patients’ acceptance of and satisfaction with HOHC. In fact, the results indicate that patients and clinical staff showed higher satisfaction and acceptance of online consultations when the Internet speed was high. However, low Internet speed can negatively influence patients’ acceptance of and satisfaction with HOHC. This indicates that there is a correlation between Internet speed and patients’ convenience with, satisfaction with, and acceptance of HOHC, which might be one of the reasons that some patients preferred in-person consultations rather than HOHC when the Internet speed was low.

Poor network services and wireless signal coverage are barriers to HOHC, which can occur because of problems with the network services and coverage themselves or because of home indoor and outdoor obstruction. This barrier affects Internet speed, which influences the quality of the HOHC; therefore, it influences patients’ acceptance of and satisfaction with HOHC. Thus, this indicates a correlation between poor networks and slow Internet speed.

Saving costs on health care services for both health care providers and patients is a key driver for adopting HOHC. It is evident that online consultations reduce service costs as well as eliminate travel costs and wait times for health care providers and patients, indicating a correlation between saving costs for patients and convenience.

Patients’ and clinicians’ training is considered a facilitator by both patients and clinicians, which enables them to use the HOHC system easily. Conversely, lack of training poses a challenge to the use of online systems and might influence users’ adoption and increase their resistance to them.

The ease of use of the online consultation system can positively influence patients’ acceptance of and satisfaction with HOHC. However, some patients with complex health conditions require complex HOHC systems, which include vital signs monitoring sensors linked to the health care provider’s data center for real-time monitoring or requiring patients to regularly report data to the health care provider. These complex systems are not easy to use, and patients might encounter technical issues and difficulties while using them. These difficulties negatively influence patients’ acceptance of and convenience with HOHC, which increase patients’ reluctance to use the technology. Therefore, the difficulty in using the system as well as the complexity of the system itself are related to patients’ complex health conditions. However, technical support, regarding technical issues during the online consultation, which patients can receive over the phone, via an online session, or by controlling their devices remotely, can reduce system difficulty.

The correlation between management and providing support to patients can be supported by the flexibility offered by online consultation in the terms of choosing a suitable time for the online treatment, documenting and tracking patients’ treatment progress in real time, as well as giving feedback to patients in a timely manner. Also, the flexibility and scheduling capabilities of online consultation systems promote convenience and compliance with treatment.

Compliance with treatment over HOHC can be more effective than in-person treatment, as it is enforced by the HOHC approach and the system as a whole. This is because patients are held accountable and are encouraged by their family members to follow the online treatment procedures. Also, compliance is aided by the convenience of the online consultation, as patients follow the treatment from the comfort of their homes at a convenient time that suits them. This indicates that compliance with treatment has a correlation with convenience and family involvement.

The *lack of organizational support* regarding the law, policy, and reimbursement are some of the most argued barriers to online consultation because health insurance companies do not fully support this type of consultation [41]. In addition, lacking support from hospitals to integrate HOHC with patients’ health records, for full record documentation, and for cross-synchronization with other system platforms is limiting the adoption of HOHC.

Accessibility to specialist care is one of the drivers that promote the use of HOHC, since it improves patients’ access to health care specialists, despite patients’ remote locations and lack of experts in their area.

Home obstructions are a barrier to online consultation. Patients are distracted by other things happening at home and the family members around them, which affects their privacy concerns.

### Internal Facilitators and Barriers (Influencers) to Home Online Health Consultation

Saving time for both health care provider staff and patients is one of the most appreciated facilitators of HOHC. Eliminating travel time is important, especially for patients in underserved areas or for nurses who perform in-person home visits. Online consultation also promotes convenience by eliminating patients’ waiting times at hospitals, outpatient units, and specialist offices.

Online consultation *resistance* often comes from patients’ lack of knowledge, unfamiliarity with technology, and resistance to change to new approaches. In this context, it should be noted that patients’ *familiarity with the system* is important in reducing their resistance. Based on the reviewed papers, users who were familiar with similar and mainstream video conferencing systems did not show resistance to online consultations. Also, *patients’ past treatment experiences* and *familiarity with clinicians* can assist in reducing technology resistance. Patients who previously had treatment for a specific health condition or patients who were familiar with the clinician who provided care for them during face-to-face consultation were more open to use HOHC systems and were encouraged to use them.

HOHC systems enable *engagement and motivation* between therapists and participants. Skilled therapists are able to engage patients in the treatment and motivate them to make healthy progress. Video conferencing can enable *excellent body language and communication* between patients and therapists, thus supporting patients’ confidence. However, lack of eye contact as well as physical and social contact (ie, *poor body language and communication*) during online consultation can be a barrier as well. In this context, *emotional support* is provided in real-time feedback, which encourages patients to commit to the treatment program.

Patients’ positive perceptions of the *system’s privacy and security*—the sense of privacy while conducting the online consultation at home—can encourage the use of HOHC. However, despite the technical effort to ensure patients’ data security and privacy, some patients show concern regarding the security of the system and their personal privacy. Patients’ *perceptions of HOHC privacy and security* are subjective; thus, it can be considered as both a facilitator and a barrier, positively or negatively influencing the view and understanding of the HOHC system.

### Effectiveness of Home Online Health Consultation and Patients’ Satisfaction

Most patients gain a high level of convenience when HOHC systems are easy to use and reduce travel time and costs, which is reflected in their satisfaction with online consultation. Also, patients are satisfied with HOHC because it is effective and convenient and provides a similar experience to face-to-face (ie, in-person) consultation. However, a small number of patients preferred face-to-face consultation for their own reasons, such as their belief that the physical presence of a health care professional would enable superior interpretation of body language and emotional expression or simply because it was their personal preference. Also, patients’ satisfaction with health care alternatives (ie, satisfaction with primary health care) has a negative influence on their attitudes toward the adoption of e-consultation and on their perception of the relative advantage of HOHC [64].

HOHC systems are effective in delivering health care services, as indicated in 44 out of 45 (98%) of the included studies. However, the use of HOHC systems with young and old patients might be difficult because young children might not engage in the online treatment [24] and patients older than 80 years might find it challenging to use them [49]. Since these results were reported only in 2 studies, and other studies with younger and older patients have been successful without reporting additional difficulties, we, therefore, do not consider age to have a significant influence on the use of HOHC.

Patients’ different health conditions, especially ones that require physical examinations, might be perceived as less accessible to clinicians by patients. Patients who underwent plastic surgery perceived that HOHC resulted in lower access to clinicians, who are to examine their surgical scars; however, the findings of that study were not significant [58]. In contrast, using HOHC with patients with burn injuries, which require a physical examination, has been successful [38]. Therefore, we can argue that the varying health conditions of patients have no significant influence on the use of HOHC.

### Practical Implications

The proposed framework (see Figure 2) of HOHC facilitators and barriers can be used for future analysis or during any development of real-time HOHC systems in a health care context. Health care providers, during the development of HOHC systems, can use the framework as a guideline to emphasize the facilitators and minimize or eliminate the barriers to ensure the delivery of effective online consultations to their patients at home. Also, this framework can be used as a clear guideline for researchers who are testing new approaches with which to implement or use HOHC to deliver care to patients with specific diseases.

### Limitations

There are several limitations to our study. First, our systematic review used the qualitative content analysis method to discover themes of facilitators and barriers to HOHC systems. This method is subject to our subjective interpretation of the findings, which may have introduced bias into the study. Also, our study is focused on a specific type of online health consultation system: a real-time HOHC system. Thus, the results may not be generalizable to all online health consultation systems, such as store-and-forward online health consultation.

A meta-analysis of the included studies was not conducted, as the number of participants in the reviewed articles varied considerably (ie, from 2 to 927 participants). Also, some studies conducted a randomized controlled trial, while other studies used cross-sectional interviews. Moreover, there could be bias in the included studies— selection bias, method bias, and reporting bias—but we did not conduct a risk-of-bias assessment.

However, despite these limitations, most of the included articles elicited similar requirements, facilitators, and barriers to HOHC, which propose a strong framework of facilitators and barriers for HOHC systems.

### Conclusions

HOHC systems can be of great benefit to patients in terms of convenience, reliability, health care availability, and cost savings. HOHC systems are tailored to meet patients’ needs, as well as to ensure effectiveness in improving patients’ well-being and satisfaction with the health care provided. Patients’ acceptance of HOHC is enforced by the facilitators, which promote effective and convenient remote treatment. However, some patients influenced by the identified barriers preferred face-to-face consultation and showed resistance to the HOHC. Future work will focus on further testing of the framework with a well-established HOHC system that receives full organizational support and a study with a large sample size of patients in order to validate the framework.

## Appendix

Multimedia Appendix 1 Aims and systems of Home Online Health Consultation (HOHC) used by each study.

Multimedia Appendix 2 Systems used for Home Online Health Consultation (HOHC).

Multimedia Appendix 3 Summary of the Home Online Health Consultation (HOHC) effectiveness and the level of evidence of each study.

Multimedia Appendix 4 Qualitative analysis of each study's facilitators and barriers.

## Abbreviations

ACM: Association for Computing Machinery
HBM: Health Belief Model
HIPPAA: Health Insurance Portability and Accountability Act
HOHC: Home Online Health Consultation
ICT: information and communication technology
MeSH: Medical Subject Headings
PRISMA: Preferred Reporting Items for Systematic Reviews and Meta-Analyses
TPB: Theory of Planned Behavior
