# Supplementary material for: Barriers and Facilitators That Influence Telemedicine-Based, Real-Time, Online Consultation at Patients’ Homes: Systematic Literature Review
Source: J Med Internet Res. 2020 Feb 20;22(2):e16407. doi: 10.2196/16407 (PMC7059083; doi:10.2196/16407)
Supplement: Multimedia Appendix 1 [file jmir_v22i2e16407_app1.docx]

## Multimedia Appendix 1. The aims and system of HOHC used by each study

Table A1-1. Aims and systems of Home Online Health Consultation used by each study

| Author | Study Aim | System Used |
| --- | --- | --- |
| Abdolahi, Bull [23] | To examine the feasibility of remote administration using The Montreal Cognitive Assessment (MoCA) via a video web-based system for individuals with movement disorders, such as Parkinson’s disease (PD) and Huntington’s disease (HD). | Patients used computers with high-speed internet access and printers to participate in this study via a web-based video conferencing system |
| Armfield, Bradford [24] | To investigate the feasibility of delivering clown therapy to children who were under care in a regional hospital or at-home care by using telemedicine service | Telemedicine, tele-clowning used on smart tablets. |
| Azar, Koliwad [25] | To examine the effectiveness, feasibility, and acceptability of an electronic Cardiometabolic lifestyle group-based intervention program | Electronic CardioMetabolic Program (eCMP),  The system consisted of wearable devices to collect physical activity data, a web portal for online materials, and a real-time encrypted videoconferencing tool for group meetings |
| Beck, Beran [18] | To explore patients with Parkinson’s disease interest in receiving a virtual call from a Parkinson’s disease specialist, to assess the system’s feasibility, and to identify barriers to enrolment. | Vidyo™ software  A video conferencing software compliant with the Health Insurance Portability and Accountability Act. |
| Benton, Heesacker [26] | To compare the effectiveness of the Therapist-Assisted Online TAO treatment therapy to traditional therapy | Therapist-Assisted Online system  Patients received online therapy via Moodle™ by using their smartphone, tablet or computer. The program provided several tools to keep participants engaged on a daily basis, such as interactive education, a weekly 10 to 15min video conference with a therapist, daily homework, task completion summary, and three weekly text messages for support and reminders. |
| Bernocchi, Vanoglio [27] | To evaluate the feasibility of a tele-surveillance and rehabilitation program which provided in-hospital rehabilitation protocols at the patient’s home. The authors also evaluated the program’s efficacy in improving patients’ functional abilities. | Home-Based Tele-surveillance and Rehabilitation (HBTR) program  A multiple platform video conference system. |
| Bull, Darwin [28] | To investigate the feasibility of conducting a remote motor assessment for patients with Huntington’s Disease and to compare the results to in-person assessment. | Vidyo  Patients installed the system on their personal PC. |
| Burkow, Vognild [29] | To assess the feasibility, acceptability, and usability of an Internet-based comprehensive, multidisciplinary pulmonary rehabilitation program at home for patients with different levels of chronic obstructive pulmonary disease (COPD) and different severity. Also, they aimed to assess patients’ outcomes and economic aspects. | Internet-based comprehensive, multidisciplinary pulmonary rehabilitation program. The system included a TV set connected to a small computer with an internet connection, a camera, and a headset to offer video conferencing. The system was controlled by a remote controller to navigate its function and to enter the daily diary. The Pulse oximetry values, and step counter values were entered manually by the patient. The system showed the diary and other values in color-coded form; yellow for normal, red for worse than normal, and green for better than normal |
| Choi and Kim [30] | To assess the feasibility of using u-health nurse management services for low-income, older patients at a reduced cost and to identify its effect on the blood pressure and level of depression of the service recipients. | Telemedicine: Videoconferencing (u-health) service,  The system provided educational materials, blood pressure (BP) monitoring devices and remote consultations at the patient’s home. Both groups were trained to measure their own BP at home, then the data sent to servers to be analysed by a clinical decision support system. Then the result was classified into colour codes based on the patient's condition (green, yellow, and red). |
| Demiris, Speedie [31] | To measure patients’ perceptions of tele-homecare before and after they have experienced it. | Tele-homecare offers a video conferencing system which includes a TV, web browser and videophone. The virtual nurse-visit was initiated by a nurse calling the patient’s phone. |
| Dimitropoulos, Zyga [32] | To assess the feasibility and acceptability of using telehealth with children with Prader– Willi syndrome (PWS) for direct intervention via video conferencing software between a child and the interventionist. | Play-Based Telehealth Intervention Program via Video conferencing system. Parents used their own computer and they were provided with a Logitech HD pro webcam (C920) for video consultation. |
| Edwards and Patel [33] | To evaluate data that were collected at various stages to conduct a comprehensive retrospective review. | Telecare was conducted via Aviva Patient Station.  Aviva units were equipped with devices such as a blood pressure cuff and stethoscope for monitoring patients’ vital signs. |
| Ehlers, Huberty [34] | To explore the acceptability and feasibility of using a tablet to deliver a group-based book club intervention. The aim of this intervention was to improve the physical activity (PA) of middle-aged women. Also, the authors evaluated the impact of the remote intervention on the social cognitive theory, which consisted of self-efficacy, self-regulation, PA benefits/barriers, social support, and self-worth. | Fit Minded program via Tablet(iPad)  The Fit Minded program focused on lifestyle and physical activity by providing a book covering topics related to memory and self-help. Further, Fit Minded provided group-based discussion aimed at social cognitive theory constructs and self-worth in a progressive manner. |
| Eslami Jahromi and Ahmadian [35] | To investigate the required infrastructure for providing teletherapy for patients with a stutter. Then the authors evaluated patients’ satisfaction with using teletherapy services and its infrastructure, to indicate their acceptance of this technology. This evaluation can be used to help policymakers to use the required infrastructure to set up such therapy. | The Skype software was used as a video conferencing platform for teletherapy sessions between the patient and the speech therapist. |
| Finkelstein, Speedie [36] | To evaluate the benefits of a TeleHomeCare program provided to patients with chronic disease (congestive heart failure, chronic obstructive pulmonary disease, or chronic wound care) after hospitalisation. | TeleHomeCare services  The program enabled communication between patients and nurses by using video conferencing, phone calls, physiological monitoring, and accessing the system website. The system used a set-top box which connected to the patient’s television set and telephone line. Also, the system used a camera which was placed on the box. |
| Finkelstein, Speedie [37] | To investigate the technical and human factors to determine the feasibility of using tele-homecare. This system provided virtual skilled nurses’ care visits to the patients’ home. | TeleHomeCare services  The program enabled communication between patients and nurses by using video conferencing, phone call, physiological monitoring, and accessing the system website. The systems used a set-top box which connects to the patient’s television set and telephone line. Also, the system used a camera which was placed on the box. |
| Garcia, Howard [38] | The authors developed an application (TeleBurn App) to enable remote treatment of patients with burn injuries. | TeleBurn App  The app provided video instruction, video conferencing, and SMS which enabled patients’ caregivers to communicate with a burn expert remotely. |
| Ghio, Boccola [39] | To investigate the use of telemedicine technology for remote care of home dialysis (automated peritoneal dialysis). | The telemedicine system used a tele-peritoneal dialysis cycler. The system supported modem-based communication between patients’ peritoneal dialysis cycler and a computer in the dialysis unit. Also, the system used communication equipment, such as camera, microphone, and computer, to support real-time and private video conferencing via ISDN line. Also, the system allowed television consultations between the physician and the patient when necessary. |
| Green, Lockhart [40] | To examine the perceived advantages and disadvantages of receiving healthy-relationships video-groups (HR-VG) at home versus at community-based organizations CBO among women living with HIV. | Internet-based healthy relationship programs were conducted via a video-conferencing system which could be accessed from the patient’s home or from community-based organizations. |
| Guillén, Arredondo [41] | The authors developed TeleHomeCare and aimed to evaluate the usability of the system. | The TeleHomeCare system has two stations (one at the patient’s home and the other at the medical centre). The home station system collects patients’ vital signs (such as blood pressure, heart rate, and EGC) and provides video conferencing between patients and doctors. The home station includes a video conferencing unit (TV, PC, and set-top-box) and the vital-signs recording unit. The medicinal centre workstation manages and accesses patients’ data and enables the care provider to communicate with their patients. The medicinal centre workstation includes a call centre, a database management system, a printer, and scanner. Both systems have a point-to-point connection between the patient’s home and hospital. |
| Harris, Freeman [42] | To examine and compare the outcome of delivering Behavioural Family Systems Therapy for Diabetes (BFST-D) for youth via tele-mental health dialogue (Skype) against clinical face-to-face delivery. The aim was to improve adherence and glycaemic control among patients (aged from 12 to 18 years old) with type one diabetes. | Skype  The intervention duration was for 12 weeks and participants completed 10 sessions of therapy. |
| Hickey, Gomez [17] | To review their initial experience of using Home Telehealth (IHT) for follow up care for burn patients | Home Telehealth (IHT)  The system used off-the-shelf video conferencing software, Vidyo™, which was used on patients’ own devices (computer or tablet) and on clinicians’ PC devices, supported with high-quality cameras. |
| Hwang, Mandrusiak [43] | To explore heart failure patients’ experiences and perspectives of using a group-based heart failure tele-rehabilitation program delivered to their home via video conferencing | The program used Adobe Connect 9.2  The program provided information booklets, demonstration sessions and introductory letters to prompt patients’ engagement. The program enabled self-mentoring, goal setting, communication, receiving and providing feedback. The program educational material was delivered as PowerPoint with voices. Before each session patients had 15 min interaction to warm up. |
| Kasschau, Sherman [44] | To assess the feasibility of a Remotely-Supervised Transcranial Direct Current Stimulation protocol for clinical use at the home of patients’ who had multiple sclerosis (MS). | Telemedicine video conferencing platform  The system included a laptop connected to a device kit, locking device, and headgear to conduct the remote treatment supported by video conferencing. |
| Mariano, Tang [45] | To evaluate patients’ changes in cognitive skills over time, to assess the feasibility and accessibility of a cognitive remediation program, and to evaluate variables associated with patients’ ability to progress successfully through the intervention program. | Tele-HomeCare via WebEx  The intervention was conducted by cognitive coaches remotely by using a standardised, computer-assisted training program (Challenging Our Minds (COM)) 3 times a week for each patient. The program incorporated a hybrid model combining “drill and practice” and “strategy-based” modes of intervention. |
| Marziali and Donahue [46] | To evaluate the feasibility of using Internet-based psychosocial and educational intervention for family caregivers of older adults with neurodegenerative disease. | Web-based video-conferencing system  The Caring for Others intervention program provided educational, psychosocial support, and problem-solving skills through the use of website features such as video conferencing, disease-specific information, private email, and a question-and-answer forum. |
| McCrossan, Morgan [47] | To assess and evaluate the feasibility of the Tele-medicine home support program. The authors examined the Tele-medicine program’s sustainability, clinical utility, and acceptance among clinicians and parents of babies with congenital heart disease (CHD). Also, the authors assessed the cost-related impact on health care resource use and the attendant cost. | Tele-medicine home support program  The telemedicine system allowed patients’ parents to answer questions related to patients’ health (feeding, weight gain, respiratory status, medications) which was assessed visually by a clinician. Clinicians then provided the necessary recommendation and assessment to parents until the next session which happened once or twice weekly |
| Melton, Brewer [48] | To report on the utilization of telemedicine technology which increased patients’ access to support-groups. The support group aimed to improve the quality of life of young adults with cancer. The authors integrated telemedicine into an existing in-person young-adult cancer group in order to address common barriers to attending the group. | Internet-based HIPAA-compliant mental health video-conferencing application.  Patients were provided with a tablet with wireless capability and were instructed to download a HIPAA-compliant video-conferencing application. They attended 6 weekly sessions of the support group (90 min each), which was facilitated by two licensed mental health professionals. |
| Peel, Russell [49] | To trial the eHAB (home video conferencing) system with elderly patients to assess the system feasibility of delivering therapy services remotely. The authors examined the healthcare practitioners’ acceptance of this system. | eHAB™ videoconferencing system.  The eHAB is a personal computer-based video conferencing system which, in this study, enabled measurement of a patient’s physical and functional performance. The system considered ease of use, which enabled clients in a home to turn it on and off by a single switch and enabled the practitioner to remotely control all aspects of the rehabilitation session. The system was used by physiotherapists, occupational therapists and speech pathologists to conduct physical consultations with patients. |
| Pietrabissa, Manzoni [50] | To examine the reasons that prompt participants to seek online psychological consultations instead of in-person consultations. Also, it aimed to explore the effect of a 30-min consultation via Facebook on participants’ motivation to seek further psychological support either online or face-to-face. Furthermore, it examined the cross-sectional associations between participants’ demographic backgrounds and their response to survey questionnaires. | Facebook |
| Portaro, Calabrò [51] | The aim of this study was to describe a telemedicine system that provides telecare for four siblings with Facio-Scapulo-Humeral Muscular Dystrophy (FSHD) disease. | The telecare system  The system provided different types of telecare services. First, psychological consultation one time a week for 30 minutes per session. Second, a monthly body mass index (BMI) assessment for 15-min per session to monitor the assigned diet. Third, cardiorespiratory rehabilitation twice a week for 40-min per session. Furthermore, neurological consultations were provided when required or in an emergency. Moreover, cardiorespiratory rehabilitation was provided by using a Virtual Reality Rehabilitation System (VRRS). |
| Rosen, McCall [52] | The authors developed a telehealth protocol to prevent patients with congestive heart failure from hospital readmission. | Telehealth was used on tablets  The program converted the standard CHF self-care protocol to a telehealth platform. Patents were provided with a tablet included software which allowed them to report on their health status. The system offered HIPAA-compliant video-conferencing software, and educational materials. Also, patients were provided with a weight scale that connected to the tablet via Bluetooth. Patients interacted with trained social workers via video conferencing weekly for education and behaviour change. |
| Tam, Man [53] | To evaluate the effectiveness and efficacy of delivering a customised tele-cognitive rehabilitation program for patients with traumatic brain injury via video conferencing. | tele-cognitive rehabilitation The program consisted of online cognitive skills training, Chinese word recognition, and memory skill retraining. The system was developed using Microsoft Windows, Microsoft Net Meeting, and Asymetrix Neuron. The program was conducted on PC machines with an internet connection. |
| Taylor, Morris [54] | To investigate technical factors that influence video conferencing quality in an in-home setting. Also, the authors assessed the impacts of these factors on clinician perception and acceptance of telehealth. The authors conducted an action research process to develop quantitative and qualitative procedures to investigate telehealth performance and users’ perceptions of it. | Vidyo application  Both groups of patients received tablet devices to perform tasks related to their health condition via video conferencing consultation. The program used the Vidyo application to perform video conferencing consultation over a Telstra 3G network and/or the Australian National Broadband Network (NBN). The program for clinicians used the Vidyo application hosted in the Flinders University data centre. |
| Thomas, McCabe [55] | To examine the efficacy of delivering Rapid Syllable Transitions (ReST) treatment for children with childhood apraxia of speech (CAS) via video conferencing at their home and compared this mode to face-to-face ReST treatment. | Adobe Connect software  Participants used their own devices connected to the internet. |
| Vijayaraghavan, O'Shea [56] | To carry out an online follow-up project to provide accessibility and cost-effectiveness diabetes intervention follow-up for patients who don't require a physical examination. Also, the online follow-up aimed to improve the efficiency of the outpatient process, to improve patients’ self-management skills and to reduce cost. | Skype |
| Vismara, McCormick [16] | To examine the use of telehealth to provide remote intervention for families to improve the skills of children with autism. | Telehealth (video-based learning modules)  The telehealth program provided two-way video conferencing and a self-guided website to host parents’ training in the comfort of their home. The system was in compliance with the Health Insurance Portability and Accountability Act (HIPAA) to ensure the privacy and security of the electronic transaction |
| Walsh and Coleman [57] | To investigate the benefit of the program on managing the health of patients with chronic disease, empowering their life and saving the cost of nursing home visits. | Telehealth home care program for Medicare patients  The telehealth system was equipped with devices to monitor patients’ vital signs, such as a blood pressure cuff. Also, the system used a video camera for video consultation. |
| Westra and Niessen [58] | To compare patients’ satisfaction in an online consultation against an in-person consultation. | Cisco Jabber: secured real-time video conferencing software. |
| Williams, Larocca [59] | To investigate the feasibility and acceptability of using a web-based model for online depression screening for college students. | Skype™. |
| Woodend, Sherrard [60] | To study the reduction in hospital readmissions after using tele-home care system, and the functional status and quality of life of patients with cardiac disease at high risk of readmission. | Telehome care system.  The intervention group was provided with 3 months of video conferencing with a nurse (held at least weekly). Also, the system provided patients with devices for weight, blood pressure and electrocardiogram measurement. |
| Wu and Keyes [15] | To assess the feasibility of a tele-exercise program with balance- impaired elderly to improve their balance and reduce their fear of falling. | Tele-Exercise  Internet-based videoconferencing device (VCD).  The exercise was delivered through a video conferencing system which allowed real-time video and audio communication between the instructor and elderly patients. The system required an internet connection, TV, and video and audio equipment to be installed in the instructor’s studio and the patients’ homes. |
| Young, Barden [61] | To conduct a qualitative study to examine the impact of Tele HomeCare (THC) on families. | Tele-HomeCare  The system monitored patients’ vital signs and provided 24/7 audio and video conferencing between nursing at hospitals and patients’ family at home over phone lines. |
| Young, Bennie [62] | To identify the changes in the quality of life of children and parents. Also, they aimed to compare the change over time between the group who received traditional care and the group who received Tele-HomeCare. | Tele-HomeCare  The system monitored patients’ vital signs and provided 24/7 audio and video conferencing between nursing at hospitals and patients’ family at home over phone lines. |
| Sorknaes, Bech [63] | The aim this study was to investigate the effect of providing home real-time teleconsultation every day for one week on patients’ hospital-readmission rate. | Real-time teleconsultations  The patient’s system was fitted in a briefcase which consist of video equipment, power switch, volume button and an alarm switch. The system was also connected to spirometer and a pulse oximeter measuring equipment. The nurse’s system consists of a computer with a built-in web camera and microphone as well as an extra screen to show patients measurements results and another computer connected to electronic medical record |
