# Supplementary material for: Barriers and Facilitators That Influence Telemedicine-Based, Real-Time, Online Consultation at Patients’ Homes: Systematic Literature Review
Source: J Med Internet Res. 2020 Feb 20;22(2):e16407. doi: 10.2196/16407 (PMC7059083; doi:10.2196/16407)
Supplement: Multimedia Appendix 2 [file jmir_v22i2e16407_app2.docx]

## Multimedia Appendix 2. Systems used for Home Online Consultation.

Table A2-1 Systems used for Home Online Consultation

| System | Articles |
| --- | --- |
| Skype™ | [35, 42, 56, 59] |
| Vidyo™ | [17, 18, 28, 54] |
| Adobe connects | [43, 55] |
| Cisco WebEx | [45] |
| Microsoft net meeting | [53] |
| Facebook. | [50] |
| Cisco Jabber. | [58] |
| Web-based video conferencing. | [16, 23, 29, 46, 48] |
| Moodle. | [26] |
| Developed their own online consultation system. | [15, 24, 25, 27, 30-34, 36-41, 44, 47, 49, 51, 52, 57, 60-63] |
