# Supplementary material for: Barriers and Facilitators That Influence Telemedicine-Based, Real-Time, Online Consultation at Patients’ Homes: Systematic Literature Review
Source: J Med Internet Res. 2020 Feb 20;22(2):e16407. doi: 10.2196/16407 (PMC7059083; doi:10.2196/16407)
Supplement: Multimedia Appendix 3 [file jmir_v22i2e16407_app3.docx]

## Multimedia Appendix 3. Summary of the HOHC effectiveness of each study

Table A3-1 Summary of the HOHC effectiveness and the Level of Evidence of each study.

| Author | Effectiveness | Level of Evidence |
| --- | --- | --- |
| Abdolahi, Bull [23] | The authors indicated that using video conferencing to administer the Montreal Cognitive Assessment remotely is feasible with individuals with mild cognitive deficits. | Level IV |
| Armfield, Bradford [24] | The result indicated extremely positive feedback from parents. The author concluded that tele-clowning enabled doctors to engage with individual patients easily and it was feasible for distance care. | Level VI |
| Azar, Koliwad [25] | The authors indicated that their finding suggested that the Electronic CardioMetabolic Program(eCMP) intervention failed to improve health-related quality of life because it did not show any significant improvement from before to after the intervention. However, eCMP showed potential in decreasing cardiometabolic risk for participants. Also, the system had good feasibility and acceptability among participants, and the authors indicated that satisfaction with eCMP was high. | Level II |
| Beck, Beran [18] | The authors indicated that there was a high demand for such a service among participants. The authors argued that convenience may be the driver for such high demand. Also, they indicated that half of the participants came from countries with limited access to neurological care. | Level II  ﻿ |
| Benton, Heesacker [26] | Therapist-Assisted, online (TAO) was a highly effective treatment. The treatment reduced patients’ anxiety symptoms, and their sense of well-being, life function, and overall mental health. | Level III |
| Bernocchi, Vanoglio [27] | The authors indicated that the program was feasible for home disease management in post-stroke patients. | Level IV |
| Bull, Darwin [28] | The authors argued that the virtual visit was feasible, and the reliability assessment of motor skills was good in comparison to in-person assessment. | Level III |
| Burkow, Vognild [29] | The result of the acceptability assessment showed that the program was well perceived by all participants and improved their health condition. | Level IV |
| Choi and Kim [30] | The authors indicated that the system changed patients’ healthy lifestyles significantly in both experimental and control groups. Also, they indicated that the depression level of the experimental group decreased significantly after the treatment, whereas it increased insignificantly in the control group. The author concluded that the u-health service was effective in controlling systolic blood pressure, but it had no effect on diastolic blood pressure. | Level III |
| Demiris, Speedie [31] | The patients had positive perceptions of tele-home care in terms of trusting the technology and saving time for the nurses | Level II |
| Dimitropoulos, Zyga [32] | The authors indicated that the program was feasible to deliver intervention for children with PWS | Level IV |
| Edwards and Patel [33] | The online treatment services replaced traditional face-to-face health care treatment. | Level IV |
| Ehlers, Huberty [34] | The women indicated that the program helped them to think positively about physical activities. | Level II |
| Eslami Jahromi and Ahmadian [35] | Patients were satisfied with the tele-speech therapy method and its infrastructure, which improved their health condition. | Level IV |
| Finkelstein, Speedie [36] | The TeleHomeCare study has demonstrated that additional visits by a skilled home healthcare nurse can be provided safely and at less expense by using virtual visits. | Level II |
| Finkelstein, Speedie [37] | The nurses completed technical quality reporting with a score of 94.7% and indicated that the virtual visit was useful as the actual face-to-face visit in 90.7% of cases. The authors indicated that patients’ attitude, vision and motor control were an important indicator of program success. | Level II |
| Garcia, Howard [38] | The authors indicated that TeleBurn™ for patients with paediatric burns helped them to heal faster, saved their caregiver travel time and cost, demonstrated faster documentation of patients’ records, enabled better compliance with the treatment and decreased clinical face to face encounters. | Level IV |
| Ghio, Boccola [39] | The result showed that the system performed well for guiding, counselling and reassuring patients and their families. The authors indicated that the system reduced the overall treatment cost. | Level VI |
| Green, Lockhart [40] | The authors indicated that the patients were interested in the remote consultation at home which mean that the system was effective. | Level II |
| Guillén, Arredondo [41] | Patients showed a high rate of system acceptance. | Level III |
| Harris, Freeman [42] | The authors concluded that tele mental health therapy (BFST-D) delivered via video conferencing (Skype) showed significant improvements in youth-and parent-reported adherence. | Level II |
| Hickey, Gomez [17] | The authors indicated that Home Telehealth provided an effective follow-up modality for selected burn patients | Level IV |
| Hwang, Mandrusiak [43] | Participants in the telerehabilitation program reported positive experiences and perceptions with the program. Also, participants indicated that the program improved their knowledge about HF self-management and modified risk factors via dietary changes and reduced alcohol intake. | Level II |
| Kasschau, Sherman [44] | The authors indicated that 100% of remotely supervised sessions were executed correctly in term of placement of electrodes, device operation and well-tolerated delivery of stimulation. | Level IV |
| Mariano, Tang [45] | The authors indicated that the system was feasible, accessible and efficacious for patients with 22q11DS. | Level III |
| Marziali and Donahue [46] | The researchers indicated that participants of online group intervention experiences were similar to the clinical -based support group. They indicated that the online intervention group had a better score in stress and burden management. | Level II |
| McCrossan, Morgan [47] | The authors concluded that the tele-medicine home support program was feasible, reliable and effective. | Level II |
| Melton, Brewer [48] | Participants were comfortable with the technology. They reported it was practical and convenient and increased their sense of connection with others and they would not attend in-person group intervention. | Level II |
| Peel, Russell [49] | The authors concluded that the eHAB had the ability to deliver remote rehabilitation services, but it faced many barriers that needed to be overcome to ensure its effectiveness | Level IV |
| Pietrabissa, Manzoni [50] | The authors concluded that Facebook chatting consultation was effective for patients with undisclosed or unresolved psychological problems and was useful for increasing awareness. | Level VI |
| Portaro, Calabrò [51] | The authors indicated that home telecare for monitoring chronic diseases is a promising management tool which provides accurate and reliable data. Also, they indicated that the program empowered patients, influenced their attitudes and behaviours, and improved their medical condition. | Level IV |
| Rosen, McCall [52] | The authors indicated that patients’ adherence to the program was excellent. Also, they indicated that patients’ admissions and readmissions were significantly lower during the intervention period. | Level IV |
| Tam, Man [53] | The authors indicated that the tele-cognitive rehabilitation model provided great flexibility of service delivery for patients and it was motivating, interactive and adaptable to their needs. | Level IV |
| Taylor, Morris [54] | The authors indicated that telehealth was effective and equivalent to or better than a home visit to provide consultation. | Level IV |
| Thomas, McCabe [55] | The authors indicated that the program was effective to deliver Rapid Syllable Transitions (ReST) treatment via video conferencing and it showed improvement for children. Also, they indicated that remote ReST treatment had similar effects to face-to-face treatment. | Level III |
| Vijayaraghavan, O'Shea [56] | The authors concluded that the system was accessible and acceptable because it provided flexibility and convenience for patients. | Level VI |
| Vismara, McCormick [16] | The finding on parent satisfaction showed that parents found the program was easy to use, the information was useful and relevant, the video session was helpful, the website video modules were useful. | ﻿ Level III |
| Walsh and Coleman [57] | This pilot project demonstrated consistent positive outcomes for all patients. | Level VI |
| Westra and Niessen [58] | The authors indicated that patients in online consultation expressed equal satisfaction to the in-person consultation. | Level II |
| Williams, Larocca [59] | The online consultation was an effective and successful alternative to the in-person clinic visit because it was convenient and enabled psychologists and psychiatrists to read patients’ body language. | Level VI |
| Woodend, Sherrard [60] | The author concluded that Telehome monitoring significantly reduced patients’ hospital readmission, visits to the emergency department and the number of days spent in the hospital for patients with angina. | Level II |
| Wu and Keyes [15] | The authors concluded that the program showed significant improvements in participates’ balance and reduction in fear of falling. | Level IV |
| Young, Barden [61] | The authors indicated that TeleHomeCare helped the family’s transition to home and enhanced families and children’s wellbeing. | Level VI |
| Young, Bennie [62] | The authors concluded that the clinical services of the TeleHomeCare were effective and able to improve the transition from hospital to home for children and parents. | Level III |
| Sorknaes, Bech [63] | The author concluded that the rea-time teleconsultation was effective as the tradition clinic intervention, but it did not significantly reduce of patients’ hospital-readmission probably because of the short trial of the teleconsultation. | Level II |
