# Supplementary material for: Barriers and Facilitators That Influence Telemedicine-Based, Real-Time, Online Consultation at Patients’ Homes: Systematic Literature Review
Source: J Med Internet Res. 2020 Feb 20;22(2):e16407. doi: 10.2196/16407 (PMC7059083; doi:10.2196/16407)
Supplement: Multimedia Appendix 4 [file jmir_v22i2e16407_app4.docx]

## Multimedia Appendix 4. Qualitative analysis of each study’s facilitators and barriers

Table A4-1. Qualitative analysis of each study's Facilitators and Barriers

| Author | Facilitators | Barriers |
| --- | --- | --- |
| Abdolahi, Bull [23] | - Saving time (commuting and waiting time) which reduced the burden on their caregiver. | - Lack of knowledge of using technology (printers and computer). - Slow internet connection, leading to poor video and audio quality. |
| Armfield, Bradford [24] | - Telemedicine services were already available at the hospital. - Clown doctors were skilled. | - Difficult to engage with very young children. |
| Azar, Koliwad [25] | - Internet access. - Training to use the system. | - No information. |
| Beck, Beran [18] | - Convenience. - Internet access. - High use of internet among participants. | - Long process to get approval for the study. - Licensure. - reimbursement. - 5 individuals withdraw for the study because of technological incompatibility e.g., old operating system. |
| Benton, Heesacker [26] | - The program provided a secure connection, privacy, encrypted text messages, and a backup plan. - Training for therapies. - Orientation as training for patients. - User familiarity with technology. - Online video session time as agreed on by both patient and therapist before the treatment. - Patient accountability to follow the treatment plan. - Patients’ engagement. - Patients received supportive or encouraging feedback regarding their involvement and progress. | - Resistance to technology from both patients and therapist. - Patients had to stay within the state of Florida during treatment to comply with state laws. |
| Bernocchi, Vanoglio [27] | - Involvement of family member (caregiver) in all stages of the program treatment. - DVD used as orientation about the importance of the program. - The program was acceptable and helpful. - The program built a good relationship with nurses and patients. | - Internet connection issue. |
| Bull, Darwin [28] | - Internet access. - Training for patients. - The program saved traveling and waiting time (time spent on waiting and traveling around 289-minute round trip). | - Poor internet connection. - Low video quality and video. - The camera captured only half of the participant’s body, which did not allow for full motor examination. |
| Burkow, Vognild [29] | - Technical training session for healthcare staff and patients. - Easy to use and learn. - Patients were provided with a user manual. - Reasonable duration of the program. - Patient’s familiarity with computer - Patient’s familiarity with health care personnel. - Providing video introduction before the discussion. - Patient’s related experiences created a sportive social environment. - Saves travel cost and time - Cheaper than outpatient’s intervention. - Providing technical support. - Available internet connection. | - Wireless issue. - Privacy issue because the intervention was done in patients living room. |
| Choi and Kim [30] | - Training patients until they knew how to use the system. - Training for nurses. - The program considered Ease of use. - Patients’ education before using the system. | - Financial difficulty affected patient eating regimen because they didn’t have money to buy food. - Drop out from the study because patients lack proper knowledge about interpreting measurement results and daily measurement which affected the patient’s mood (only one female in the study) - Patients’ dependency on a medical institution for a treatment service and prescription. - Patients’ lack of a sense of need for a management-oriented service. |
| Demiris, Speedie [31] | - Nurse’s understanding of patients’ medical problem. - Patients were satisfied talking to the nurse over the television as talking in person. - The program was easy to use. - The program improved the patients’ general health. - The program saved time for the nurses. - Patients believed that the program saved their time. - Patients believed that the program helped them to contact the nurse easily. - The program reduced the costs for the health care agencies. - TeleHomeCare was convenient in delivering health care. - Patients trusted the equipment to work properly. | - Patients believed that the program couldn’t save their money. - Patients believed that the program lacked physical contact during a TeleHomeCare visit. |
| Dimitropoulos, Zyga [32] | - Training for interventionist. - Training for parents(caretakers). - Interventionist followed strict treatment procedures which were provided as manual for them. - Introducing the program to the children and their family before the treatment. - Parents provided with a manual to help them during the online session. - Availability of camera, computer, and internet connection. - Interventionist use of positive feedback and encouragements to engage parents and patients. | - Some technical issues related to the quality of the video due to internet connection. - Audio issue due to internet connection. - Difficulty to place the camera at a good angle. - Lack of a dedicated room for conducting the intervention. |
| Edwards and Patel [33] | - Saving traveling cost. - Sustainability of networks. - Organizational support. - Saving the cost of health services. | - Reticence to adapt to new technology. - Concerns about liability and reimbursement. - Issues relate to lighting, getting used to audio delay or motion artifacts, and “glitches” in the image. |
| Ehlers, Huberty [34] | - Patients believed that books were useful and helped them in improving their physical activity. - Patients preferred e-book on the iPad. - Tutorial on using the tablet before the intervention. - Patients believed that the iPad was easy to use and user-friendly. | - Patients had time and scheduling conflicts. - Patients felt disconnected during the video due to the lack of social presence. - Some patients believed that some features of the program were not easy to use (blogs). - Patients’ digital literacy and lack of experience with the tablet. - Delay in audio resulted in participants talking over each other. - Some patients spent time on resolving technical difficulties. - Patients lacked attention during the meeting because they were doing other home tasks such as cooking, watching TV, answering the phone and talking with other members of the family. - Some patients didn't like wearing accelerometers during the study time because it was not convenient, and it was not suitable for all occasions. |
| Eslami Jahromi and Ahmadian [35] | - Skype™ was very popular among Iranian users. - Skype uses low internet speed connection. - Skype was easy to use. - Skype provides security. - The author promised patients information privacy and confidently. - All session was not recorded - Saving travel and waiting time, and travel cost. | - Low internet speed connection (n=24). - Lack of eye contact (n=24). - Hard to understand and express emotion. - Sens of Informality during each session (﻿lack of seriousness). |
| Finkelstein, Speedie [36] | - Training on using the system. - Patients were physically and cognitively able to use the system equipment. - Technically functional home environment (a telephone and television, the close proximity of telephone line to the television and adequate lighting). | - Patients were concerned about using the computer and the internet. - Issues related to provider acceptance and cost and reimbursement policy. |
| Finkelstein, Speedie [37] | - Nursing knowledge, enthusiasm, and actions toward and about telemedicine were important indicators of the program success. - Patients’ attitude, vision and motor control were important indicators of program success. - Flexibility in modifying the equipment, software interfaces, and information presentation enabled adaptation to the patient’s needs. - Training sessions were provided to participants. | - Some patients’ concern about using the equipment prevented them from participating in this study. |
| Garcia, Howard [38] | - Demonstrated faster documentation of patients recorded. - Enabled better compliance with the treatment. - Saved their caregiver travel time and cost. - Helped patients to heal faster. - Better management. - Real-time assessment. - Compliance with Health Insurance Portability and Accountability Act (HIPPAA). - Ensured patients data security Involved expert during the app development. | - The app was under development, which led to some drop out of the study. |
| Ghio, Boccola [39] | - Training the family to use the system the treatment. | - Patients often find dialysis stressful. - Some technical and logistical issues related to long-time installation, lost connection, and defects in new equipment. |
| Green, Lockhart [40] | - The program was convenient as the treatment was conducted from the comfort of home. - Technology availability. | - Some patients showed concerns about privacy when someone living in the home can be seen by others in the video conference. - Some patients showed concerns about privacy in disclosure of their health status to family members who did not know about it. - Distractions of surrounding home environment. - Lack of high-speed internet access could be a major barrier to expanding access to the program. |
| Guillén, Arredondo [41] | - The program offers ease of use to improve acceptance with people with no technology background. - Patients’ interaction with the system was similar to the common interaction with the TV set, which increased system acceptance. - The system interface was suitable to create a virtual presence between patients and doctor. - The system ability to function over low bandwidth connection enables it to work fine over HFC or ADSL communication networks. - Patients training. - Patients’ feeling of presence and privacy during the online session was positive. | - Reimbursement and legal issues. - Lack of patients’ training and knowledge regarding technology use. - The high cost of the system hardware. - The high cost of the system operation. |
| Harris, Freeman [42] | - Fast internet connection. - Convenience. - Saving travel cost and time. - Easy to use skype. - Improving accessibility to specialist - Ethical, legal and practice implications considered during the consultation. | - Slow internet connection. |
| Hickey, Gomez [17] | - High-resolution video camera. - Vidyo™ provided a secure, HIPAA compliant, and private platform for video conferencing. - Patients used their own devices. - Security and privacy at home were the patient’s responsibility. - Saving travel time and cost (188 miles per patient ranged from 4-822 mile, saved average travel expenses of $108.50 per patient). - A telehealth coordinator created Health accounts for patients and completed a test call to troubleshoot patients’ devices, software, and/or internet connection prior to the physician-patient interaction. | - Poor video quality. - Low audio volume. - Some patient needed to restart their compute.r |
| Hwang, Mandrusiak [43] | - Improved patients’ health outcomes. - Enabled access to care and eliminated transportation, and travel time. - Enabled social support between family and other - Easy to use the system. - The program was Convenient and lowered family member traveling burden. - Easily accessible, safe - The program was structured, which facilitated patients’ adherence to the treatment. - The program improved patients’ knowledge about heart failure, self-management, and modified risk factors via dietary changes and alcohol intake. - The program provided an appropriate level of supervision from a distance. | - Learning new technology and fear of unknown. - Technical issues related to the quality of the connection, which resulted in the delay in audio, auditory fading, video freezing, small video window, and absence of video. - Technical issues related to internet connection dropout. |
| Kasschau, Sherman [44] | - Having clear protocol and guidelines for treatment. - Training for patients. - Video and manual instruction for patients were provided. - Patients had no contraindicated health conditions or environmental distractions. - Patients were capable using a laptop computer. - Patients were able to tolerate a tDCS session. - Patients’ commitment to the scheduled session time for the duration of the study. - Control over the patient’s laptop for troubleshooting. - Saving travel time and cost. - Ease of use. | - No information. |
| Mariano, Tang [45] | - The system was child-friendly. - Difficult levels of tasks (treatment) were removed. - Older patients performed better. - Trained and experienced coaches. | - Internet access (one of the participants dropped out of the program because he/she internet issue). |
| Marziali and Donahue [46] | - Training for participants. - Ease of use. - Privacy. - Security. - Building trust between participants. | - No information. |
| McCrossan, Morgan [47] | - High video quality. - The system allowed accurate reading of vital signs. - System ease of use. | - Technical problem; loss of internet connection. |
| Melton, Brewer [48] | - The flexibility of group scheduling and the program could be accessed from any place. - Saved travel time and travel cost. - Reminder email one or two days before each session. - Patients were comfortable with the technology, the group, and the video call. - The system was practical and convenient, increased patients’ sense of connection with others - Patients indicated that they would not attend in-person group intervention because the system was effective. | - Delayed delivery of the tablet, - Patients missed some of the sessions because they had planned engagements, they forgot, they had a medical appointment, or they had a medical emergency. |
| Peel, Russell [49] | - Designed for ease of use. - Enabled practitioner to remotely control all aspects of the rehabilitation session. | - Patient’s low mobility. - Patient’s complex social problems. - Patient’s low hearing and vision. - Patient’s cognitive impairment. - The system weight and size were challenging for patients to carry it around the home and to store it in a safe place at home. - Patients required assistance from a third person to use the system. |
| Pietrabissa, Manzoni [50] | - The economic convenience of the program. - Patients’ sense of shame associated with requesting an in-person consultation helped in participating in the online session. - Patients’ curiosity to use Facebook for consultation. - Patients’ previous experience with the therapist encouraged them to participate in study. - Facebook was easy to use. - The treatment was free, easy to access, and a short-term commitment, which encouraged them to participate in the study. - Patient’s location far from psychologist centre encouraged them to participate in the study. | - Patient’s location close to the psychologist centre prevented them from participating in the study. |
| Portaro, Calabrò [51] | - Caregiver training to use the system. - Easy to use. - Continuous monitoring of patients’ vital signs. - Telemedicine protocol to monitor patients based on their health condition severity. | - No information. |
| Rosen, McCall [52] | - Trained social workers. - The program was designed for patients with low health literacy and was very easy to use. | - No information. |
| Tam, Man [53] | - The program offered great flexibility of service delivery for patients. - The program was motivating, interactive. - Adaptable to patients needs. - The therapist had control over client’s computer screen. - Health insurance coverage for this type of services was provided. | - Depends on the quality of the internet connection so if there is any problem with the connection it will affect the intervention quality. - The home environment may be hard to control to reduce noises and distraction. - The system should be user-friendly, and the program should be explained to the family member - Technological glitches. - Technology resistance from nurses - Lack of integrated documentation of patients recorded. |
| Taylor, Morris [54] | - The program was simple and easy to use. - Saving time for participants. - The program saves travel time. - The program increased time efficiency. - The program worked well over the NBN internet connection. | - Poor 3G signal coverage. - Home construction affected the signal strength. - Bad weather conditions affected the signal strength. - Some patients were uncomfortable with the technology. |
| Thomas, McCabe [55] | - Save travel time. - Easy to use. - Training for children before the treatment (one or 2 online training session). - Training for physician. - Convenient. - Motivated children via the game - Internet speed (that 3 out five had speed download above 50Mbps and 2 had below 4 Mbps). | - Audio latency. - Difficulty establishing the audio connection. - Video freezing. - Slow internet speed. - Some patients had no internet access. |
| Vijayaraghavan, O'Shea [56] | - Using readily available system improved acceptability. - Using widely-used software improved acceptability. - Saving time and cost for patients. - Saving cost for the healthcare provider. | - Lack of internet connection. - Patients’ lack of confidence to use the technology. - Older people above 60 years old had less uptake of the system. - Some patients preferred face-to-face follow-up. |
| Vismara, McCormick [16] | - The program offered flexible scheduling. - Training for parents. - Training for therapies. - The system was easy to use. - The program eliminated travel expenses. - The program offered convenience and flexibility in online learning, and the amount of information that could be observed, stored and shared electronically. - The program Followed privacy guideline. | - The program lacked cross-synchronization with other popular platforms. - The website and/or features were hard to navigate, such as having to click on several links before accessing the desired outcome. |
| Walsh and Coleman [57] | - Health insurance coverage for this type of services. | - Technological glitches. - Technology resistance from nurses. - Lack of integrated documentation software. |
| Westra and Niessen [58] | - Patients in the online group showed less waiting time and spent less time on their appointment than the control group. - Saving travel time. | - Some patients required assistance to install the software. - The program was not easy to use. - Patients indicated that they felt their physician was unable to physically examine their surgical scars due to lack of adequate attention. - Physician’s low communication skills. |
| Williams, Larocca [59] | - The program had clear audio and video quality. - The program promoted convenience. - The program enabled psychologists and psychiatrists to read patients’ body language cues. - The program was easy to use and offered 256-bit data encryption. - Patients were familiar with the system. | - Security concerns - Internal barriers (Patients stigma) - Patients’ lack of depression awareness. - Patients’ lack of time (Too busy/I forgot to complete the online consultation) - Patients’ inflexible schedule. - The study was a one-time opportunity. |
| Woodend, Sherrard [60] | - The program was easy to use. - Training for participants to use the system and the equipment before the treatment. | - Patients had difficulty in using some parts of the system. - Patients had difficulty in using c-phone (resulted in poor video conferencing quality). |
| Wu and Keyes [15] | - Training for participants. - Internet access. - Technical support. - Ease of use. - The program offered convenience (no need to go to the gym) - The program promoted the feeling of privacy for the participants when exercising at home. - The program promoted social interactions among the participants. - Feedback in a timely manner between participants and instructors. | - Connection problems and audio problems. - Hardware problems (such as failed camera, microphone, or audio circuitry). - Remote control multiple buttons were confusing and difficult to see and manipulate, which caused unintentional problems related to audio communication. - Lack of echo cancelling capability in the program. - Camera’s focal length didn't allow for capturing the full body of the participants, but it was changed to wide-angle in second session. |
| Young, Barden [61] | - Training for participants. - Availability (the system was available 24/7). | - Video quality issue - Lack of licensing for physicians across jurisdictions. - Reimbursement for this type of service. |
| Young, Bennie [62] | - A Phone line at home. - The ability of the family member to provide care to their child. | - No information. |
| Sorknaes, Bech [63] | - Nurses’ technological skills. | - No information. |
